# Interactome of Glyceraldehyde-3-Phosphate Dehydrogenase Points to the Existence of Metabolons in *Paracoccidioides lutzii*

**DOI:** 10.3389/fmicb.2019.01537

**Published:** 2019-07-09

**Authors:** Kleber Santiago Freitas e Silva, Raisa Melo Lima, Lilian Cristiane Baeza, Patrícia de Sousa Lima, Thuany de Moura Cordeiro, Sébastien Charneau, Roosevelt Alves da Silva, Célia Maria de Almeida Soares, Maristela Pereira

**Affiliations:** ^1^Laboratório de Biologia Molecular, Instituto de Ciências Biológicas, Universidade Federal de Goiás, Goiânia, Brazil; ^2^Laboratório de Bioquímica e Química de Proteínas, Departamento de Biologia Celular, Universidade de Brasília, Brasília, Brazil; ^3^Núcleo Colaborativo de Biossistemas, Instituto de Ciências Exatas, Universidade Federal de Jataí, Goiás, Brazil

**Keywords:** GAPDH, *Paracoccidioides*, protein-protein interaction, metabolon, interactome

## Abstract

*Paracoccidioides* is a dimorphic fungus, the causative agent of paracoccidioidomycosis. The disease is endemic within Latin America and prevalent in Brazil. The treatment is based on azoles, sulfonamides and amphotericin B. The seeking for new treatment approaches is a real necessity for neglected infections. Glyceraldehyde-3-phosphate dehydrogenase (GAPDH) is an essential glycolytic enzyme, well known for its multitude of functions within cells, therefore categorized as a moonlight protein. To our knowledge, this is the first approach performed on the *Paracoccidioides* genus regarding the description of PPIs having GAPDH as a target. Here, we show an overview of experimental GAPDH interactome in different phases of *Paracoccidioides lutzii* and an *in silico* analysis of 18 proteins partners. GAPDH interacted with 207 proteins in *P. lutzii*. Several proteins bound to GAPDH in mycelium, transition and yeast phases are common to important pathways such as glycolysis and TCA. We performed a co-immunoprecipitation assay to validate the complex formed by GAPDH with triose phosphate isomerase, enolase, isocitrate lyase and 2-methylcitrate synthase. We found GAPDH participating in complexes with proteins of specific pathways, indicating the existence of a glycolytic and a TCA metabolon in *P. lutzii*. GAPDH interacted with several proteins that undergoes regulation by nitrosylation. In addition, we modeled the GAPDH 3-D structure, performed molecular dynamics and molecular docking in order to identify the interacting interface between GAPDH and the interacting proteins. Despite the large number of interacting proteins, GAPDH has only four main regions of contact with interacting proteins, reflecting its ancestrality and conservation over evolution.

## Introduction

*Paracoccidioides* is a dimorphic fungi and the genus includes species that cause paracoccidioidomycosis (PCM). The disease is endemic in Latin America and prevalent in Brazil (Brummer et al., 1993). The genus *Paracoccidioides* comprises five distinct species *Paracoccidioides brasiliensis, Paracoccidioides lutzii, Paracoccidioides ameri*cana, *Paracoccidioides venezuelensis*, and *Paracoccidioides restrepiensis* (Muñoz et al., 2016; Turissini et al., 2017). The mycelium phase grows in the environment or in culture at 22°C. The fungus undergoes differentiation into the yeast form at 37°C in culture or within the host lungs and then disseminates into several organs (Brummer et al., 1993). The morphogenetic shift from mycelia to yeast deserves special attention once it denotes a virulence strategy of the pathogen to establish the infection (Rooney and Klein, 2002). The PCM treatment is arduous (Travassos et al., 2008) and the most common drugs used are based on azoles, sulfonamides and amphotericin B and they present a considerable degree of toxicity. Moreover, the period of treatment can be extended to years in some cases (Bocca et al., 2013).

Glyceraldehyde-3-phosphate dehydrogenase (GAPDH) is an essential glycolytic enzyme, also well known for its multitude of functions within cells. Therefore, it is categorized as a moonlight protein. GAPDH is widely explored virtually in all organisms and it has been shown to play vital roles in eukaryotic metabolism (Seidler, 2013), production of exporting vesicles (Tisdale, 2001), regulating proteins (Kornberg et al., 2010), related to the cell cycle and apoptosis (Tarze et al., 2007) and protecting cells against reactive oxygen species (ROS) (Hara et al., 2005). GAPDH was also found to be forming glycolytic multiprotein complexes in *Saccharomyces cerevisiae* (Brandina et al., 2006), with the heat shock protein (HSP) family in hepatitis B virus (Liu et al., 2009) and in plants (Bustos and Iglesias, 2003), besides being exported and contributing to *Streptococcus pyogenes* virulence (Jin et al., 2011). GAPDH has been intensely investigated in *Paracoccidioides*. It is differentially expressed in several approaches such as copper and zinc deprivation in *P. lutzii* (Parente et al., 2013; de Oliveira et al., 2014), up regulated in *P. brasiliensis* yeast cells (Rezende et al., 2011), acting as adhesin (Barbosa et al., 2006), present in the secretome of mycelia and yeast cells (Weber et al., 2012) and interacting with malate synthase (de Oliveira et al., 2013). As a moonlight macromolecule, GAPDH interacts with a large variety of proteins, forming multiprotein complexes that play unique roles within cells (Brandina et al., 2006; Ferreira et al., 2013). Interactions among proteins have been described by innumerous techniques such as immunoprecipitation (Ferreira et al., 2013), yeast two-hybrid and pull down (de Oliveira et al., 2013), tandem affinity purification (Xu et al., 2010), affinity chromatography (Wong et al., 2004) and Blue Native PAGE (BN-PAGE) (Brandina et al., 2006).

The study of protein-protein interactions (PPIs) adds knowledge related to protein function and helps to characterize protein complexes and the pathway they are involved, giving insight into new roles of proteins or adding more information to biological known processes. GAPDH establishes interactions with other proteins (Brandina et al., 2006; Liu et al., 2009; Kornberg et al., 2010; Araiza-Olivera et al., 2013; Ferreira et al., 2013) and due to the importance of GAPDH over cell dynamics, here we show an overview of PPIs of GAPDH in *P. lutzii* in order to explore its functions and participation in different metabolic processes.

Here, we show an overview of experimental GAPDH interactome in different phases of *P*. *lutzii* and an *in silico* validation of 18 proteins that bound to GAPDH through the experimental approaches. GAPDH interacts with 207 proteins in *P. lutzii*. We modeled the GAPDH 3-D structure, performed molecular dynamics and molecular docking in order to identify the interacting interface between GAPDH and the interacting proteins. Moreover, we found that molecular dynamics significantly improved GAPDH 3-D model and that GAPDH has four main regions of contact with interacting proteins. Several proteins bound to GAPDH in mycelium, transition and yeast phases are common to important pathways such as glycolysis and (tricarboxylic citric acid) TCA. The interaction between GAPDH and triose phosphate isomerase, enolase, 2-methylcitrate synthase and isocitrate lyase was validated by co-immunoprecipitation assay. GAPDH of *P. lutzii* is a moonlight protein and its interactome shows that GAPDH participate in a large variety of biological processes in the pathogen.

## Materials and Methods

### *P. lutzii* Mycelium and Yeast Culture Maintenance

*Paracoccidioides lutzii* (ATCC-MYA-826) was used in all experiments in the present study. This isolate is the most representative in the region where this work was performed and responsible for most of the PCM cases in central-west of Brazil. It was cultivated on Fava-Netto solid medium (1.0% w/v peptone, 0.5% w/v yeast extract, 0.3% w/v proteose peptone, 0.5% w/v beef extract, 0.5% w/v NaCl, 4% w/v glucose, and 1.4% w/v agar, pH 7.2) (Fava-Netto and Raphael, 1961) at 22°C for the growth of *P. lutzii* mycelium phase or 37°C for the growth of the yeast phase. The media was renewed every 7 days.

### *P. lutzii* Mycelium to Yeast Transition

*Paracoccidioides lutzii* mycelium to yeast transition was performed in Fava-Netto liquid medium (Fava-Netto and Raphael, 1961) by a change in the culture temperature from 22°C to 37°C (Da Silva et al., 1994). *P. Lutzii* mycelium cells were previously grown in liquid medium for 48 h at 22°C before the temperature shift to 37°C. The samples were collected at 72 h of growth in order to perform morphological monitoring through a Neubauer chamber.

### *P. lutzii* Protein Extraction Procedure

Mycelium cells were collected after 14 days of growth, mycelium-to-yeast transition cells after 3 days post temperature shift and yeast cells after 3 days of growth. Each sample phase was submitted to total protein extraction. Cells were collected by centrifugation at 10,000 × *g* for 10 min at 4°C and washed in sterile phosphate buffered saline (PBS; 1.4 mM KH_2_PO_4_, 8 mM Na_2_HPO_4_, 140 mM NaCl, and 2.5 mM KCl at pH 7.2). The cells were then resuspended in a buffer with 20 mM Tris–HCl at pH 8.8 and 2 mM CaCl_2_ and added a protease inhibitor mix (GE Healthcare, Uppsala County, Uppsala, Sweden) in order to prevent protein degradation. The samples were conditioned in proper tubes with glass beads and applied to a bead beater apparatus (BioSpec, Bartlesville, OK, United States) for 5 cycles of 30 s each, in order to disrupt cells and expose proteins to the solution. Samples were kept on ice to avoid protein denaturation. Then, the samples were centrifuged at 10,000 × *g* for 15 min at 4°C. The supernatant was collected and the protein concentration was determined using Bradford reagent (Sigma-Aldrich, St. Louis, MO, United States). Bovine serum albumin (BSA) was used as standard.

### *P. lutzii* GAPDH Heterologous Expression and Recombinant Protein Purification

Glyceraldehyde-3-phosphate dehydrogenase recombinant protein was obtained as described by Barbosa et al. (2006). Briefly, we used the TOPO-pET-100-GAPDH construct in order to produce recombinant GAPDH. The recombinant protein synthesis started by the addition of isopropyl-β-D-thiogalactoside (IPTG) (Sigma-Aldrich) to a final concentration of 1 mM. BL21 cells expressing recombinant *P. lutzii* GAPDH were incubated with 1 μ/mL lysozyme and underwent subsequent sonication on ice. For the recombinant GAPDH with his-tag at the N-terminal end purification, we used the nickel-nitrilotriacetic acid resin (Ni-NTA) (Invitrogen, Carlsbad, CA, United States). For 1 mL of lysate, we used 250 μL of resin. The lysate was incubated with the resin on ice under gentle agitation for 2 h. Then the resin was washed 5 times with native wash buffer (50 mM Na_2_HPO_4_, 20 mM imidazol, pH 8.0) in order to eliminate contaminants. The recombinant GAPDH bound to the resin was eluted with native elution buffer (50 mM Na_2_HPO_4_, 250 mM imidazol, pH 6.0). The purity and size of the protein were assessed by a 12% sodium dodecyl sulfate-polyacrylamide gel electrophoresis (SDS-PAGE) followed by Coomassie staining.

### *P. lutzii* GAPDH Pull Down Assay

Nickel-nitrilotriacetic acid resin pull down assay is an affinity chromatography procedure that uses a bait protein, *P. lutzii* GAPDH in our case, immobilized and incubated with a protein source containing putative protein preys. The assay was performed in native conditions and the results included direct and indirect associations between bait and prey proteins. Firstly, recombinant GAPDH was immobilized onto Ni-NTA resin following the purification assay without the elution step. Then, 300 μg of *P. lutzii* cell lysate containing the protein extract was incubated for 3 h on ice and under gentle agitation. Next, the column containing bait and prey proteins was washed with native wash buffer 5 times to reduce unspecific interactions or contaminants and then, the complex bait-preys was eluted with native elution buffer. The eluted sample underwent tryptic digestion and the digested peptides were separated further via NanoUPLC-MS^E^ and analyzed using a nanoACQUITY system (Waters Corporation, Milford, Manchester, United Kingdom) in order to identify the proteins that possibly interacted with GAPDH.

A control sample was prepared similarly but 300 μg of *P. lutzii* protein extract was incubated with Ni-NTA without the immobilized GAPDH and the proteins identified in both experiments were excluded from the results.

### Blue Native PAGE Approach

Blue Native-PAGE was performed according to the protocol established by Schägger et al. (1994) with some modifications. The samples loaded in the gels were *P*. *lutzii* mycelium, mycelium-to-yeast and yeast water-soluble proteins. The samples (300 μg of proteins) were dissolved in 10% (w/v) glycerol and 50 mM Bistris/HCl at pH 7.0. A 5–18% (w/v) polyacrylamide gradient gel was casted and a gel buffer (150 mM Bistris/HCl, 1.5 M aminocaproic acid at pH 7.0), a cathode buffer (50 mM tricine, 15 mM Bistris/HCl, 0.02% Coomassie blue G-250, at pH 7.0) and an anode buffer (50 mM Bistris/HCl at pH 7.0) were used to conduct native electrophoresis. The electrophoresis was performed on a vertical Hoefer SE600 ruby apparatus (GE Healthcare) at 12°C, with a starting voltage of 100 V until the loaded samples were inside the stacking gel, then a constant current limited to 300 V and 15 mA were applied.

### Co-immunoprecipitation

The assay was performed according to Araiza-Olivera et al. (2013) with some modifications. Briefly, target antibodies were incubated with protein A sepharose 4B (Invitrogen, Waltham, MA, United States) for 3 h. Then, the solution antibodies and resin was incubated with 3 mg/mL of total protein extract for 3 h. Washing steps were performed with wash solution, 10 m mM tris at pH 7.4, 1% Triton and 1 mM EDTA (ethylenediaminetetraacetic acid). Elution was performed with 0.2 M glycine at pH 2.6 and equal amount of 0.2 M tris at pH 8.0. Eluted samples underwent SDS (sodium dodecyl sulfate)-PAGE followed by Western blot.

### STRING Database Analysis

The STRING (Search Tool for the Retrieval of Interacting Genes/Proteins) database focuses on the prediction and unification of PPIs, direct and indirect associations, in order to cover even organisms where experiments are unavailable. Here, the accession numbers of identified proteins were analyzed for their interactions using STRING database version 9.1^1^ (Szklarczyk et al., 2011). In order to perform the analysis we included active interaction sources such as text mining, experiments, databases, and co-expression with the highest confidence score (0.900).

### In-Gel Enzymatic Digestion

The in-gel digestion was performed in accordance with (Rezende et al., 2011). The Coomassie blue-stained spots were manually excised from the gels, washed twice with MilliQ H_2_O, dehydrated in 100 μL of acetonitrile (ACN) and dried in a speed vacuum (Eppendorf, Hamburg, Germany). Following, the reduction with 30 μL of 10 mM dithiothreitol (DTT) solution (GE Healthcare, Piscataway, NJ, United States), incubated in a dry bath at 56°C for 1 h and alkylated with 30 μL of 55 mM iodoacetamide (GE Healthcare, Piscataway, NJ, United States) for 45 min at room temperature in the dark. The supernatant was removed, and the gels were washed with 100 μL of a 25 mM ammonium bicarbonate solution by vigorous mixing for 10 min, and dehydrated twice in 100 μL of a 25 mM ammonium bicarbonate and 50% (v/v) ACN solution, vortexed for 5 min and centrifuged. The gel pieces were dried, and a 0.01 μg/μL trypsin solution (Promega, Madison, WI, United States) was added, followed by rehydration on ice at 4°C for 10 min. The supernatant was removed, and 25 μL of 25 mM ammonium bicarbonate solution was added to the gel pieces, followed by incubation at 37°C for 16 h. Then, the supernatant was placed into a clean tube. Fifty microliters of 50% (v/v) ACN, 5% (v/v) trifluoroacetic acid (TFA) solution was added to the gel pieces. The samples were vortexed for 10 min and the supernatant was then combined with the solution retrieved after digestion. The samples were dried in a speed vacuum, and the peptides were solubilized in 10 μL MilliQ H_2_O. The concentration and purification was performed using a pipette tip (ZipTips^®^C18 Pipette Tips, Milipore, Bedford, MA, United States).

### Digestion of Protein Extracts

Enzymatic digestion of proteins was processed according to Murad et al. (2011) and Murad and Rech (2012) with some modifications. Briefly, 50 μg of total protein was added to 10 μL of 50 mM ammonium bicarbonate pH 8.5, in a microcentrifuge tube. Then 25 μL of RapiGEST^TM^ SF Surfactant (0.2% v/v) (Waters, Milford, MA, United States) was added, and the sample was vortexed and incubated in a dry bath at 80°C for 15 min. Following the reduction of disulfide bonds with 2.5 μL of 100 mM dithiothreitol (DTT) (GE Healthcare, Piscataway, NJ, United States) at 60°C for 30 min and alkylation of cysteine with 2.5 μL of 300 mM iodoacetamide (GE Healthcare, Piscataway, NJ, United States) for 30 mim at room temperature in the dark. The proteins were digested with 10 μL of trypsin 0.05 μg/μL (Promega, Madison, WI, United States) at 37°C in dry bath for 16 h. To precipitate the RapiGEST, 10 μL of a 5% trifluoroacetic acid solution (Sigma-Aldrich, St. Louis, MO, United States) was added, the samples were vortexed, and incubated for 90 min at 37°C in a dry bath, and centrifuged at 18,000 × *g* at 4°C for 30 min. The supernatant was dried in a speed vaccum apparatus (Eppendorf, Hamburg, Germany). All obtained peptides were suspended in 50 μL of a solution containing 20 mM of ammonium formate and 100 fmol/μL of phosphorylase B (Waters Corporation, Manchester, United Kingdom) (MassPREP^TM^ protein).

### NanoLC-MS^E^ Acquisition

Nanoscale LC separation of tryptic peptides was performed using a ACQUITY UPLC^®^M-Class system (Waters Corporation, United States) equipped with a XBridge^®^Peptide 5 μm BEH130 C18 300 μm × 50 mm pre-column; Trap, 2D Symmetry^®^5 μm BEH100 C18, 180 μm × 20 mm column and Peptide CSH^TM^ BEH130 C18 1.7 μm, 100 μm × 100 mm analytical reversed-phase column (Waters Corporation, United States). The peptides obtained from the digestion of proteins in the pull down assay were separated using a gradient of 11.4, 14.7, 17.4, 20.7, and 50% of ACN and 0.1% (v/v) formic acid, with a flow rate of 2000 μL/min. For samples obtained from the gel pieces, 4 μL full loop injections were initially transferred with an aqueous 0.1% formic acid solution to the pre-column. After desalting and pre-concentration, the peptides were eluted from the pre-column to the analytical column and separated with a gradient of 3–45% mobile phase B (0.1% formic acid in ACN) over 40 min at a flow rate of 2000 μL/min, followed by a 10 min rinse with 99.9% of mobile phase B.

The lock mass was used for calibration of the apparatus, using a constant flow rate of 0.5 μL/min at a concentration of 200 fmol of GFP ([Glu1]-Fibrinopeptide B human (Sigma-Aldrich, St. Louis, MO, United States). Mass spectrometry analysis was performed on a Synapt G1 MS^TM^ (Waters, United States) equipped with a nanoelectrospray source and two mass analyzers: a quadrupole and a time-of-flight (TOF) operating in TOF V-mode. Data were obtained using the instrument in the MS^E^ mode, which switches the low energy (6 V) and elevated energy (40 V) acquisition modes every 0.4 s. Samples were analyzed from three replicates.

### Data Processing and Protein Identification

The mass spectrometer data obtained from the LC-MS^E^ analysis were processed using the ProteinLynx Global Server version 3.0.2 (Waters, Manchester, United Kingdom). The data were subjected to automatic background subtraction, deisotoping and charge state deconvolution. The processed spectra were searched against *Paracoccidioides Pb*01 protein sequences (Broad Institute)^2^^,^^3^ . The mass error tolerance for peptide identification was under 50 ppm. The protein identification criteria also included the detection of at least two fragment ions per peptide, five fragments per protein and the determination of at least one peptide per protein; the identification of the protein was allowed with a maximum 4% false positive discovery rate in at least three technical replicate injections. The searches were performed with fixed modification of carbamidomethyl-C, and variable modifications were phosphorylation of serine, threonine and tyrosine. One missed cleavage site was allowed. The minimum repeat rate number for each protein in all replicates was two. Protein tables generated by ProteinLynx Global Server were merged and the dynamic range of the experiment was calculated using the software program MassPivot v1.0.1. The peptide and protein tables were compared using the Spotfire v8.0 software, suitable graphics were generated for all data as previously described (Murad et al., 2011). Microsoft Excel (Microsoft, United States) was used for table manipulations. UniProt^4^ and Pedant on MIPS^5^ databases were used for functional classification. NCBI database was employed for annotation of uncharacterized proteins^6^. The mass spectrometry proteomics data have been deposited to the ProteomeXchange Consortium via the PRIDE (Vizcaíno et al., 2016) partner repository with the dataset identifiers PXD008253, PXD008308, PXD008309, and PXD008317.

### Preparation of 3D Structures, Molecular Dynamics and Molecular Docking

The I-TASSER server^7^ modeled GAPDH and interacting proteins. The I-TASSER modeling is based on templates of homologous proteins with experimental structure available on the PDB database (Yang et al., 2015). All proteins under study had their three-dimensional structure assessed for quality using the MolProbity server^8^ (Cheng et al., 2007).

In order to obtain a more stable structure and similar to the native model, the target protein (GAPDH) was submitted to molecular dynamics (MD). The MD was performed using the software GROMACS 4.5.5, AMBER force field (ff99SB-ILDM), with the presence of explicit solvent (water TIP3P) in order to get a more stable protein structure (Pronk et al., 2013). In the first stage of MD, the overall free-energy was minimized in order to remove conflicting contacts and the process was completed only when the tolerance of 1000 kJ/mol was not exceeded. Seeking the balance of the thermodynamic variables of the system, a 100 ps simulation of NVT (amount of substance in moles, volume and temperature) and NPT (amount of substance in moles, pressure and temperature) simulation were applied. For the NVT phase, where there is variation in the pressure of the system, the temperature was set to 300 K by the thermostat and the velocities were calculated through Maxwell’s equations. For the NPT simulation, the volume is allowed to vary and the pressure was maintained constant by the Parrinello-Rahman barostat (Iannuzzi et al., 2003; Hutter, 2011).

Next, the protein was submitted to a simulation of 100 ns, 300 K, 1 atm and time interval of 2 fs, without restriction of the protein conformation (Pronk et al., 2013). MD analysis of GAPDH trajectories was performed by root mean square deviation (RMSD) in relation to the initial structure by the *gromos* algorithm (Daura et al., 2004). The g_cluster program (GROMACS package) was used to determine the conformations that were most frequent during the simulation. We used a cut-off of 0.3 nm in order to distinguish the conformational sets based on the RMSD profile. The cluster analysis and the RMSD allow the analysis of the protein profile throughout the simulation and the most representative conformational mode was selected to undergo molecular docking (Pronk et al., 2013).

We used the GRAMM-X protein-protein anchor server^9^ to determine the best protein complex conformations between GAPDH and interacting proteins (Tovchigrechko and Vakser, 2006). After the assembly of the complexes, the next step was to identify the amino acids involved in the interaction. We used the KFC2 server^10^ to recognize all residues from the interaction interface from GAPDH and interacting protein (Zhu and Mitchell, 2011).

We used the CoCoMAPS^11^ server after the most frequent GAPDH residues involved in the interactions were identified. CoCoMAPS enables the analysis and visualization of the interface of interaction in protein complexes by making use of intermolecular contact maps in order to identify hot spot residues (Vangone et al., 2011). After identifying the interacting residues we used Pymol molecular visualization program to highlight the GAPDH regions most frequently involved in the interactions.

### Identification of Nitrosylation Target Signal

We used the software GPS-SNO 1.0 (Xue et al., 2010) for the prediction of nitrosylation sites. A nitrosylation signal peptide comprises a short sequence of amino acids with a cysteine residue, normally placed in the middle of the sequence. As peptides with similar amino acid sequences may present similar structures, a substitution matrix is used in order to compute similarity between two peptide sequences. The results undergo clusterization steps and a score is generated along with a cut-off value and the determination in which cluster the signal peptide was identified.

## Results and Discussion

### Overview of GAPDH Interacting Proteins

**FIGURE 1:**
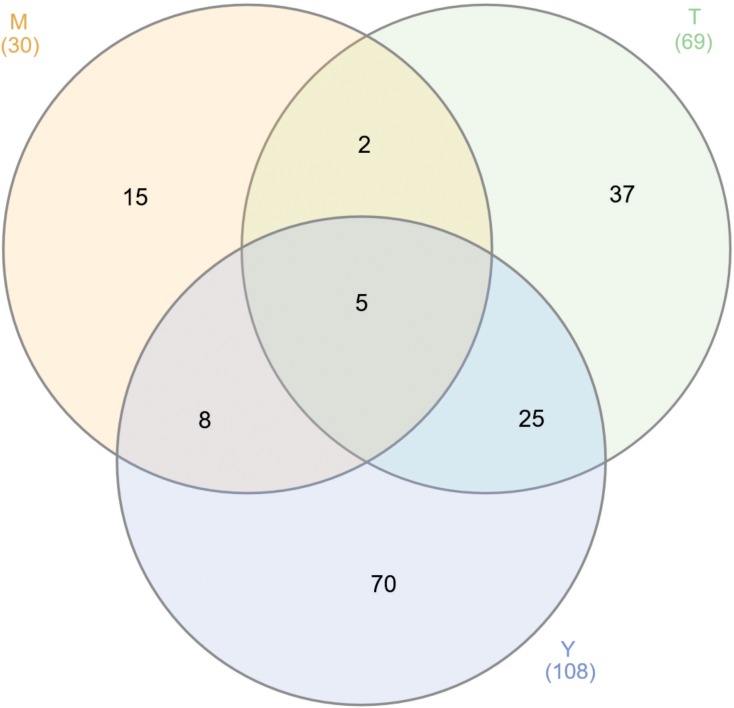
Venn diagram of GAPDH interacting proteins. We found 207 proteins interacting with GAPDH through two different approaches and performed independently for the three stages of *P. lutzii* life cycle. The numbers on the diagram represent the number of proteins interacting with GAPDH in mycelium (M), mycelium-to-yeast transition (T) and yeast (Y) *P. lutzii* phases.

The PPI assays performed here identified 207 GAPDH interacting proteins. Among those interactions, 30 (14.5%) occurred in mycelium, 69 (33.3%) in mycelium-to-yeast transition and 108 (52.2%) in *P. lutzii* yeast cells. Some of the GAPDH interactions were identified in more than one morphological phase of the fungus (Figure 1). The difference in GAPDH binding patterns among those phases results from two main factors. Firstly, certain proteins are differentially expressed according to the life cycle of the pathogen (Rezende et al., 2011) and secondly, some of the interactions may be lost during experimental procedures (Hayes et al., 2016).

**FIGURE 2:**
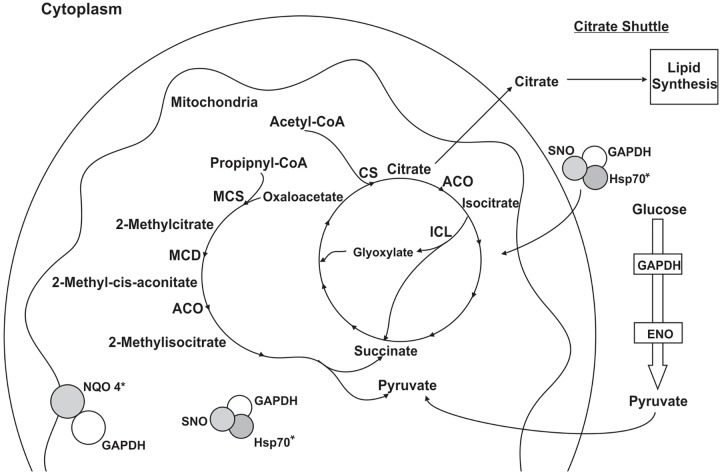
Schematic model of the metabolic processes involving GAPDH through PPIs in *P. lutzii* mycelium phase. All proteins shown in the figures interacted with GAPDH as a result of the chromatographic and BN-PAGE approaches. GAPDH (glyceraldehyde 3-phosphate dehydrogenase), SNO (nitrosylation), CS (citrate synthase), ACO (aconitase), ICL (isocitrate lyase), MCS (2-methylcitrate synthase), MCD (2-methylcitrate dehydratase), HSP60 (heat shock protein 60), HSP70 (heat shock protein 70), NQO4 (NAD(P)H:quinone oxidoreductase type IV) and ENO (enolase). Asterisks indicate proteins identified as GAPDH partners by both the chromatographic and BN-PAGE approaches.

**FIGURE 3:**
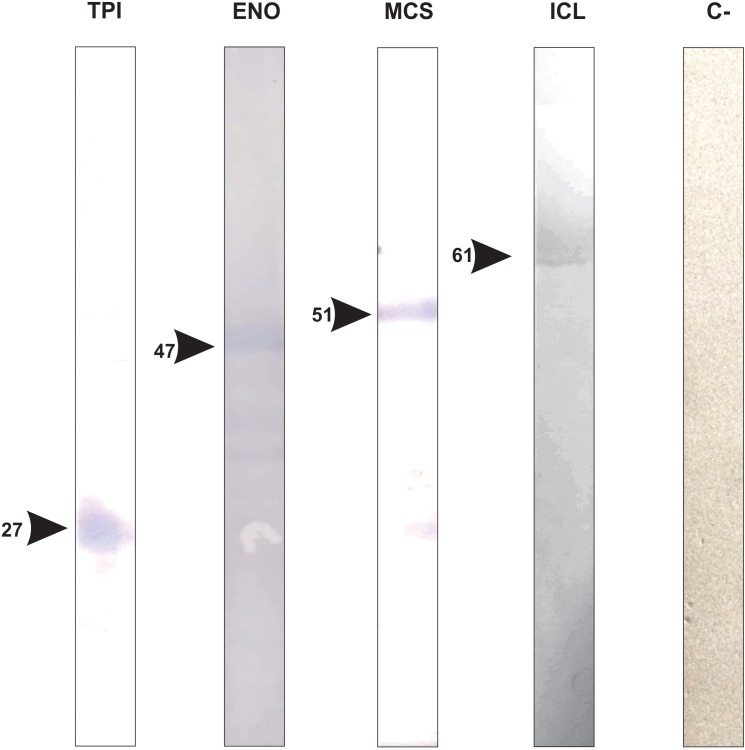
Validation of GAPDH interacting proteins by co-immunoprecipitation assay. The figure shows the confirmation of GAPDH being complexed with proteins that we found as a result of chromatographic and co-immunoprecipitation assays TPI (triose phosphate isomerase), ENO (enolase), MCS (2-methylcitrate synthase), ICL (isocitrate lyase) and C-(negative control).

According to the pull down assay, 18 proteins from *P. lutzii* mycelium phase bound to the recombinant GAPDH (Supplementary Table S1). Their biological classification includes metabolism (28%), energy (28%), protein synthesis (11%), protein fate (5%), cell rescue, defense and virulence (5%), biogenesis of cellular components (5%) and hypothetical proteins (18%) (Figure 2). The multiprotein complex identified by BN-PAGE in mycelium phase, included proteins related to metabolism (14%), energy (29%), protein synthesis (7%) and protein fate (50%) (Supplementary Table S2). Interestingly, enolase (ENO) and isocitrate lyase (ICL) interaction with GAPDH was found in both PPI approaches. We performed a co-immunoprecipitation assay to validate the complex formed by GAPDH and ENO and ICL (Figure 3).

**FIGURE 4:**
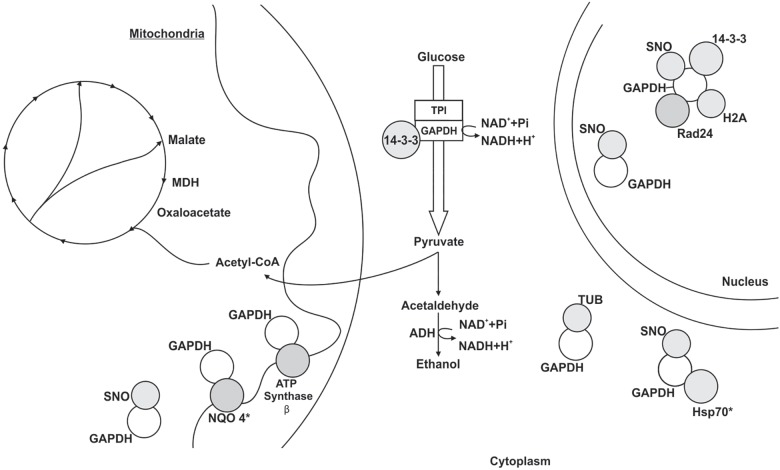
Schematic model of the metabolic processes involving GAPDH through PPIs in *P. lutzii* mycelium-to-yeast transition. All proteins shown in the figures interacted with GAPDH. GAPDH (glyceraldehyde-3-phosphate dehydrogenase), SNO (nitrosylation), MDH (malate dehydrogenase), TPI (triosephosphate isomerase), NQO4 (NAD(P)H:quinone oxidoreductase type IV), tubulin α-1 chain (TUB), HSP70 (heat shock protein 70), histone H2A (H2A) and ADH (alcohol dehydrogenase). The figure also shows the proteins 14-3-3, Rad24 and ATP synthase β. Asterisks indicate proteins identified as GAPDH partners by both the chromatographic and BN-PAGE approaches.

The pull down assay, regarding mycelium-to-yeast transition, identified 54 proteins interacting with GAPDH (Supplementary Table S3). They were related to metabolism (19%), energy (7%), cell cycle and DNA processing (11%), transcription (7%), protein synthesis (32%), protein fate (9%) and hypothetical proteins (15%). For the BN-PAGE assay (Supplementary Table S4), GAPDH interacting proteins were related to metabolism (33%), energy (17%), cell cycle and DNA processing (22%), protein fate (17%) and hypothetical proteins (11%) (Figure 4). NAD(P)H:quinone oxidoreductase (NQO4) and an isoform of HSP70 interacted with GAPDH in both assays (Supplementary Tables S3, S4). We found six proteins bound to GAPDH that were up-regulated during mycelium-to-yeast transition in comparison to mycelium and yeast phases in *P. brasiliensis* (Rezende et al., 2011) and those proteins were malate dehydrogenase (MDH), ATP synthase β, two variants of HSP70, HSP90, alcohol dehydrogenase (ADH) and adenosylhomocysteinase (Figure 2). These findings show that GAPDH is likely to play important roles during *Paracoccidioides* spp. mycelium-to-yeast transition. Due to its probable role in substrate channeling, the interaction between GAPDH and triose phosphate isomerase (TPI) (Sweetlove and Fernie, 2018) were validated by a co-immunoprecipitation assay (Figure 3).

**FIGURE 5:**
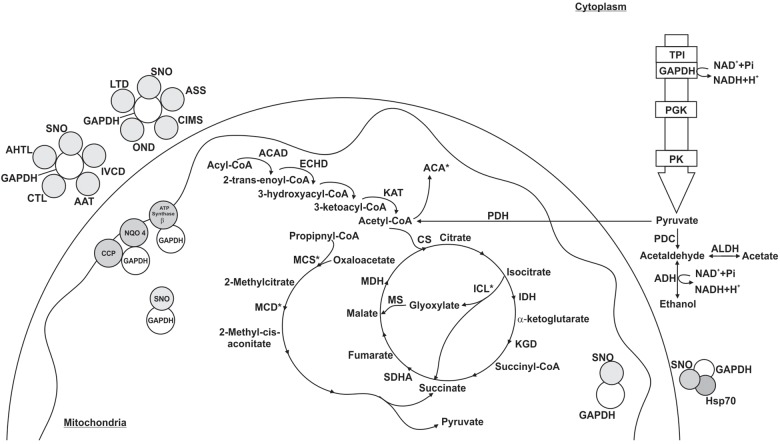
Schematic model of the metabolic processes involving GAPDH through PPI in *P. lutzii* yeast phase. All proteins shown in the figures interacted with GAPDH. GAPDH (glyceraldehyde-3-phosphate dehydrogenase), SNO (nitrosylation), ACAD (acyl-CoA dehydrogenase), ECHD (enoyl-CoA hydratase), KAT (3-ketoacyl-CoA thiolase), CS (citrate synthase), IDH (isocitrate dehydrogenase), ICL (isocitrate lyase), KGD (dihydrolipoyl dehydrogenase), SDHA (succinate dehydrogenase flavoprotein subunit), MDH (malate dehydrogenase), MCS (2-methylcitrate synthase), MCD (2-methylcitrate dehydratase), TPI (triosephosphate isomerase), PGK (phosphoglycerate kinase), PK (pyruvate kinase), PDH (pyruvate dehydrogenase E1 component subunit β), PDC (pyruvate decarboxylase), ADH (alcohol dehydrogenase), ALDH (aldehyde dehydrogenase), ACA (acetyl-CoA acetyltransferase), AHTL (*o*-acetylhomoserine thiol-lyase), CTL (cystathionine γ-lyase), AAT (aspartate aminotransferase), IVCD (isovaleryl-CoA dehydrogenase), ASS (argininosuccinate synthase), CIMS (cobalamin-independent methionine synthase), OND (oxidoreductase 2-nitropropane dioxygenas), LTD (L-threonine 3-dehydrogenase), CCP (cytochrome c peroxidase) and NQO4 (NAD(P)H:quinone oxidoreductase type IV). The figure also shows the proteins HSP70 (heat shock protein 70) and ATP synthase β. Asterisks indicate proteins identified as GAPDH partners by both the chromatographic and BN-PAGE approaches.

The pull down approach identified 88 target proteins interacting with GAPDH in the yeast phase. We found proteins related to metabolism (23%), energy (18%), cell cycle and DNA processing (8%), protein synthesis (39%), protein fate (11%) and hypothetical proteins (1%) (Supplementary Table S5). For the BN-PAGE approach (Supplementary Table S6), we found 26 GAPDH interacting proteins related to metabolism (19%), energy (23%), cell cycle and DNA processing (4%), protein synthesis (11%), protein fate (12%), cellular transport (4%), signal transduction (4%) and hypothetical proteins (23%) (Figure 5). GAPDH interaction with ICL, 2-methylcitrate synthase (MCS), 2-methylcitrate dehydratase (MCD), acetyl-CoA acetyltransferase (ACA) and HSP90 were found in both PPI approaches performed (Supplementary Tables S5, S6).

Among the proteins that interacted with GAPDH, 15 were up regulated in the yeast morphological phase of *P. brasiliensis* compared to the mycelium and transition phases (Supplementary Table S5) (Rezende et al., 2011), showing that GAPDH has important roles regarding specific activities played by *P. lutzii* yeast cells in a variety of biological processes (Figure 5). In addition, interaction between GAPDH and MCS was validated by co-immunoprecipitation (Figure 3).

### GAPDH Integrates Metabolons in Substrate Channeling

Glyceraldehyde-3-phosphate dehydrogenase is a moonlight protein with a multitude of functions (Barbosa et al., 2006; Rezende et al., 2011; Weber et al., 2012; de Oliveira et al., 2013, 2014; Parente et al., 2013) due to interactions with other proteins. Here, GAPDH interacts with several proteins related to energy metabolism via glycolysis, TCA, respiration, glyoxylate and methylcitrate pathways. It has been shown that mycelium *P. lutzii* cells use aerobic metabolism to obtain energy while the yeast cells use anaerobic metabolism (Felipe et al., 2005). We found GAPDH interacting with proteins from aerobic metabolism such as ENO, citrate synthase (CS), aconitase (ACO) and (NQO4) with proteins from anaerobic metabolism, such as ICL. Other studies demonstrate that among several moonlight function of GAPDH is to be a sensor of cellular stress (Colell et al., 2009; Tristan et al., 2011) and control of the aerobic and anaerobic metabolic shift (Schuppe-Koistinen et al., 1994).

Glyceraldehyde-3-phosphate dehydrogenase interacts with four glycolytic enzymes, ENO (Supplementary Tables S1, S2), TPI (Supplementary Tables S4, S5), pyruvate kinase (PK) and phosphoglycerate kinase (PGK) (Supplementary Table S5). The interactions between GAPDH and ENO and TPI were identified in the affinity chromatography assay (Supplementary Table S1), BN-PAGE (Supplementary Table S2), co-immunoprecipitation (Figure 2) and in the STRING PPI database. GAPDH interacting with PK was identified in the affinity chromatography assay and in the STRING database. Several PPI studies corroborate our findings regarding GAPDH interacting with glycolytic enzymes ENO (Kumar et al., 2004; Ferreira et al., 2013), TPI (Stephan et al., 1986; Liaud et al., 2000; Kumar et al., 2004; Graham et al., 2007; Araiza-Olivera et al., 2013; Menard et al., 2014), PGK (Prabhakar et al., 1999; Tomokuni et al., 2010; Araiza-Olivera et al., 2013; Wan et al., 2015) and PK (Kumar et al., 2004; Araiza-Olivera et al., 2013; Ferreira et al., 2013; Wan et al., 2015; Das et al., 2016). Coupling catalytic function of enzymes, such as GAPDH and other glycolytic enzymes, affords optimization of regulation and combined functionality of the reactions. They associate in order to accomplish their metabolic functions (Auger et al., 2015) and to channel pyruvate toward TCA cycle, establishing a tangible connection between glycolysis and TCA.

Protein-protein interactions studies in *S. cerevisiae* through BN-PAGE and immunoprecipitation approaches resulted in 13 proteins associated in a macromolecular complex (Brandina et al., 2006). They found three proteins from the glycolytic pathway, including ENO and GAPDH. PPI studies on *Escherichia coli* found GAPDH interacting with ENO and other glycolytic enzymes (Kumar et al., 2004; Ferreira et al., 2013). In *S. cerevisiae*, glycolytic enzymes associate among themselves and with actin filaments in order to stabilize and protect enzymes in a glycolytic metabolon (Araiza-Olivera et al., 2013). This is the first time a metabolon is identified in *P. lutzii* and we found GAPDH interacting with actin cytoskeleton protein (VIP1) in the pull down assay (Supplementary Table S3), which strengthens the idea of the existence of a glycolytic metabolon in *P. lutzii*. The interaction between GAPDH and TPI is a coherent example of substrate channeling because the product of the TPI reaction is the substrate of GAPDH. Metabolons increase the concentration of enzymatic intermediates locally and decrease the amount of enzyme needed to maintain the intermediate flux in a pathway (Sweetlove and Fernie, 2018). Hence, the final product is directed in a scaffold manner to specific subcellular spots and the metabolon prevents loss of those intermediates.

Glyceraldehyde-3-phosphate dehydrogenase interacts with several enzymes of TCA. We also found GAPDH bound to two enzymes from the pyruvate dehydrogenase complex (pyruvate dehydrogenase E1 component subunit β and dihydrolipoyl dehydrogenase; Supplementary Table S5). These enzymatic complex converts pyruvate into acetyl-CoA and the multiprotein complex formed with GAPDH could drive acetyl-CoA into its destiny by substrate channeling. GAPDH and TCA enzymes have been found to form protein complexes with glycolytic enzymes and mitochondrial membrane carriers in *S. cerevisiae* (Brandina et al., 2006).

We found GAPDH bound to MDH (Figure 4) and the presence of MDH among up regulated proteins in *P. brasiliensis* mycelium-to-yeast transition is noteworthy (Rezende et al., 2011). Another study detected, through a pull down assay, potential interactions between *Porphyromonas gingivalis* proteins and *Streptococcus oralis* recombinant GAPDH (Maeda et al., 2013). MDH was among the proteins investigated. Moreover, they showed that *P. gingivalis* MDH is a surface associated protein and the interaction between the recombinant GAPDH and MDH inhibited *P. gingivalis* and *S. oralis* biofilm formation. In addition, GAPDH is on the cell surface of several organisms, including *P. gingivalis* where it is related to the regulation of host invasion (Tribble et al., 2006) and *P. brasiliensis* where it is involved in fungal adhesion to extracellular proteins (Barbosa et al., 2006; Weber et al., 2012). These results indicate that an interaction between GAPDH and MDH may play a role in the host-pathogen relationship.

Glyceraldehyde-3-phosphate dehydrogenase interacted with isocitrate dehydrogenase (IDH) (Supplementary Table S5). IDH catalyzes the conversion of isocitrate into α-ketoglutarate and is regulated by acetylation. A deacetylase family protein named sirtuin (SIRT) stimulates its activity (Someya et al., 2010). Interestingly, SIRT is negatively regulated by GAPDH through nitrosylation (Kornberg et al., 2010). GAPDH and IDH interaction has been shown by other study and they found GAPDH and IDH in a multiprotein complex that used thioredoxin as protein bait (Kumar et al., 2004). Another protein found in this thioredoxin multiprotein complex was succinate dehydrogenase flavoprotein subunit (SDHA) (Supplementary Table S5), which corroborates our findings regarding the pull down assay.

The proteins ICL, MCS (Supplementary Tables S1, S5, S6) and MCD (Supplementary Tables S2, S5, S6) interactions with GAPDH were identified in both PPI approaches. The former takes part in the glyoxylate cycle and the other two enzymes belong to the methylcitrate pathway. ICL and MCD were up regulated in *P. lutzii* yeast cells, which utilize anaerobic metabolism driving two-carbon sources to feed glyoxylate cycle and three-carbon sources, such as propionate, to feed methylcitrate cycle. GAPDH was found in trypanosome peroxisomes (Opperdoes and Borst, 1977) and here we found GAPDH interacting with peroxin-19. Peroxins takes part in the recognition of proteins with peroxisomal or glyoxysomal targeting signals and consequently mark them to be transported into peroxisomes. This explains that *P. lutzii* GAPDH may be found in peroxisomes allowing its interaction with proteins from the glyoxylate cycle. Moreover, it has been shown through affinity chromatography and *in silico* approaches that glyoxylate cycle enzymes interact with GAPDH in *P. lutzii* (de Oliveira et al., 2013).

Regarding cellular respiration, we found GAPDH interacting with NQO4 (Supplementary Tables S1, S3–S5), ATP synthase β (Supplementary Tables S3, S5) and cytochrome c peroxidase (Supplementary Table S5), showing a probable regulation of ATP intracellular levels. Ferreira et al. (2013) showed that GAPDH may have high affinity for ATP-binding domains. They found GAPDH interacting with ATP-binding proteins and ATP synthase subunits α and β. The latter was also found in studies regarding multiprotein complexes related to the protein thioredoxin on *E. coli* (Kumar et al., 2004). The relation between GAPDH and cellular energy levels has been investigated in *S. pyogenes* where mutants incapable of export GAPDH to the cellular surface, increased the intracellular ATP concentration significantly compared to wild strains. Moreover, they showed that up regulation of GAPDH also led to up regulation of ATPases genes (Jin et al., 2011). A study performed on *E. coli* showed through immunoprecipitation and pull down assays that GAPDH interacts with ATP synthase β (Ferreira et al., 2013). They concluded that the interaction between those proteins indicates an additional process for the regulation of ATP dependent processes. In *S. cerevisiae*, NQO4 and GAPDH were in the same multiprotein complex (Gavin et al., 2002) and another group found GADPH interacting with cytochrome c oxidase in bovine heart tissue (Ramzan et al., 2013). These interactions altogether show that GADPH influences intracellular ATP levels.

### GAPDH Participates in Nitrosylation Processes

Glyceraldehyde-3-phosphate dehydrogenase takes part in protein regulation via gasotransmitters (Mustafa et al., 2009). Hydrogen sulfide (H_2_S), nitric oxide (NO) and carbon monoxide (CO) mediate cellular processes regulating several protein functions (Nguyen et al., 2011). Some proteins interact via thiol and disulfide bonds, normally at cysteine residues. A study on gasotransmitters showed that around 25% of cellular GAPDH is sulfhydrated and exerts functions apart from the glycolytic pathway (Hara et al., 2005). This is one of the reasons why GAPDH has several partners, being categorized as a moonlight protein. GAPDH acts as a nitrosylase (Hara et al., 2005) and also undergo nitrosylation (Kohr et al., 2014), increasing considerably the number of interacting partners. Interestingly, many of the proteins that were identified in the present work as interacting with GAPDH has a target signal for nitrosylation (Gould et al., 2013) (Supplementary Table S7). To support the hypothesis that GAPDH is a nitrosylase of mitochondria proteins, it has been shown that nitrosylated proteins that are expressed when GAPDH is overexpressed were down regulated after GAPDH knockdown in heart cells of mice (Kohr et al., 2014).

The nitrosylation features a covalent addition of nitric oxide to a cysteine thiol in a target protein and exerts regulatory function from bacteria to mammalian cells (Anand and Stamler, 2012). Co-immunoprecipitation experiments in mice cardiac cells showed that GAPDH regulates TCA enzymes (Kohr et al., 2014), including ACO that we found to be one of the GAPDH interacting protein in *P. lutzii* mycelium cells (Figure 2). Another study performing the same methodological experiment on *S. cerevisiae* also found GAPDH and TCA enzymes in the same complex (CS and MDH) (Kohr et al., 2011; Gould et al., 2013), showing that interactions among those enzymes must be playing important roles in regulatory pathways, metabolite channeling and maintaining a link between glycolysis and TCA (Brandina et al., 2006). In addition, the glyoxysome enzyme, ICL, undergoes nitrosylation and it has been shown that its activity is reversibly reduced after nitrosylation (Morisse et al., 2014).

The presence of GAPDH in mitochondria is related to its potential function as a nitrosylase of several mitochondrial protein targets (Kohr et al., 2014). We found GAPDH interacting with proteins from β-oxidation, among them acyl-CoA dehydrogenase (ACAD) (Doulias et al., 2013), 3-ketoacyl-CoA thiolase (KAT) (Supplementary Table S5) and enoyl-CoA hydratase (ECHD) (Supplementary Tables S4, S6) (Piantadosi, 2012). Nitrosylation also targets proteins related to amino acid metabolism, such as the gasotransmitter protein argininosuccinate synthase (ASS). We found this protein interacting with GAPDH in the pull down approach (Supplementary Table S5) and a large scale PPI study also found ASS bound to GAPDH (Wan et al., 2015). The biosynthesis pathway of the amino acid arginine is regulated by the nitrosylation of ASS, which inhibits the activity of this enzyme. The NO from the nitrosylation process acts as an important signaling molecule, and the regulation takes place at cysteine residues present at the surface of the interacting proteins (Hao et al., 2004).

Cell cycle proteins interacted with GAPDH in both approaches employed (Supplementary Tables S3, S4). The protein 14-3-3 has a multitude of functions, including regulation of signaling pathways and cell cycle. GAPDH and the protein 14-3-3 interacted in the BN-PAGE approach (Supplementary Table S4). The interaction between GAPDH and 14-3-3 proteins have been shown in several organisms such as plants (Bustos and Iglesias, 2003), human (Pozuelo Rubio et al., 2004) and hydra (Pauly et al., 2007). In the latter, this interaction increased sensitivity to inhibition by ATP, thus being regulated, which maintain safe levels of energy within cytoplasm (Pauly et al., 2007). In *P. brasiliensis*, 14-3-3 protein has been reported to be an adhesin (Assato et al., 2015), to be related to apoptosis (Silva Jde et al., 2015) and to be involved in fungus pathogenesis (Marcos et al., 2016). All those activities are also played by GAPDH (Barbosa et al., 2006; Tarze et al., 2007) in *Paracoccidioides* spp. and other microorganisms such as yeast (Carmona-Gutierrez et al., 2010).

Glyceraldehyde-3-phosphate dehydrogenase is present in the nucleus of cells (Sawa et al., 1997) in several organisms and one of the many functions played by this protein is DNA repair (Ferreira et al., 2013). We found GAPDH interacting with DNA damage checkpoint protein rad24 (Supplementary Table S4), which is related to DNA repair prior to mitosis. Some studies have classified rad24 as belonging to the 14-3-3 protein family (Ozoe et al., 2002). GAPDH, rad24 and the protein 14-3-3. We also found GAPDH interacting with histone H2A in the pull down approach (Supplementary Tables S3, S5). Histone H2A is also related to DNA repair when phosphorylated (Jakob et al., 2011). Several studies reported the relationship between GADPH and histone H2A expression, which depends on a redox status that could be guaranteed by GAPDH in multiprotein complexes (Shi and Shi, 2004; Dai et al., 2008; He et al., 2013).

Here, GAPDH interacted with ADH and aldehyde dehydrogenase (AHDH) (Supplementary Tables S4, S6). ADH acts in the interconversion of ethanol to acetaldehyde, producing NADH, while GAPDH glycolytic reaction promotes the reduction of NAD^+^ to NADH. Thus, both proteins within a multiprotein complex are able to provide or deprive the reducing equivalent NADH in response to metabolic changes. ADH is immunogenic in several microorganisms, including *Candida albicans* (Bertram et al., 1996) and *P. brasiliensis* (Dantas et al., 2009). GAPDH and ADH have been found in the same protein complex in large-scale PPI approaches (Gavin et al., 2002). A study performed on *S. cerevisiae* found GAPDH and ADH interacting in two complexes along with other proteins that we have also identified in our approaches, such as proteins from the cell division control (Supplementary Table S3) and the HSP (Supplementary Tables S3, S4) family (Gavin et al., 2002). A different large-scale study on *Drosophila melanogaster* identified 556 protein complexes and GAPDH and ADH were among the interacting proteins (Guruharsha et al., 2011).

The interaction between GAPDH and ADH or GAPDH and ALDH is found in other organisms as well (Gavin et al., 2002; Wong et al., 2004; Guruharsha et al., 2011; Ferreira et al., 2013). ADH is overexpressed in *P. brasiliensis* yeast phase undergoing anaerobic conditions (Rezende et al., 2011), which leads to a metabolic shift and ethanol production via pyruvate (Felipe et al., 2005). Ethanol is used to damage tissue and facilitates host invasion besides producing energy (Grahl et al., 2011; Luong et al., 2015). ALDH catalyzes the oxidation of aldehydes and participates in detoxification of such compounds (Marchitti et al., 2008). Through the activity of ALDH, pathogens evade osmotic, oxidative and other cellular stressors besides host immune response (Singh et al., 2004). The interaction between GAPDH and ALDH may optimize host invasion and protection against stress conditions (Abdul et al., 2018).

We found several HSPs complexing with GAPDH in *P. lutzii* (Supplementary Tables S2–S6). HSPs act as molecular chaperones and control protein biogenesis and correct folding (Craig et al., 1994). The protein HSP60 is mostly mitochondrial but is present in the cytoplasm in lower levels. It is related to the correct folding of proteins, stabilization of unfolded proteins and assembly of multimeric complexes (Soltys and Gupta, 1999). HSP70 and its isoforms bind to incompletely folded proteins, such as polypeptides on ribosomes and proteins that undergo transportation from cytosol into mitochondria, such as GAPDH (Murphy et al., 2014). HSP90 acts on modulation of protein activity (Craig et al., 1994).

The BN-PAGE approach was performed to study the HSP machinery involved in hepatitis B infection and it was found a protein complex with GAPDH, HSP60, HSP70, and HSP90 (Liu et al., 2009). The authors stated that proteins presented in the complexes they analyzed influenced the viral infection. HSPs play a key role maintaining the protein complex functional and HSP 70 and 90 could be candidates for antiviral therapy once they are related to the host-pathogen interaction (Liu et al., 2009).

### Molecular Dynamics Improved GAPDH 3-D Model

After predicting three-dimensional structure of GAPDH through the I-TASSER server, the stereochemistry of the three-dimensional model were assessed by the Ramachandran plo (Supplementary Figures S1A,B). We performed GAPDH molecular dynamics in 100 ns. Supplementary Figure S1A shows that before the MD 77.1% residues were within favored regions and 90.8% of the residues were within allowed regions. The figure also shows that 9.22% of the amino acids in the initial conformation were at unfavorable regions. Supplementary Figure S1B shows that after MD, 89.0% residues were within favored regions and 97.3% of the residues were within allowed regions. Moreover, only 2.7% of the residues were within unfavorable regions and it is mostly likely that those regions are not present in the binding and catalytic site of GAPDH and may refer to unconserved regions. The number of residues with atoms in unfavorable angular positions decreased sharply. Before the MD, we had 31 residues in a forbidden position and after the MD, this number lowered to 9. Ramachandran plots consistently showed that GAPDH structure is refined after MD (Supplementary Figures S1A,B).

Protein stability is an important trait for protein interactions. Hydrogen bonds, hydrophobic and electrostatic interactions are key to determine the stability of a protein and a protein complex. Rational design of peptides and drugs in order to inhibit PPIs tries to improve stabilizing interactions between protein and ligand. We used RMSD to plot the analyses of MD trajectories to establish the equilibration period and quality of the biomolecular simulations besides clustering similar conformations. For RMSD at a value of 0.4 nm, the variation of the simulations becomes more stable (Supplementary Figure S1C). According to the trajectories of MD simulations, we identified at least seven conformational clusters. Cluster analysis showed that the structure of the first three clusters had a more stable conformation. Moreover, the first cluster remained stable for a longer period than the other clusters (Supplementary Figure S1D). The Molprobity score of the total structure improved from 2.78 to 1.62, and the clash-score from 6.44 to 0. The sharp reduction of those scores indicate the number of structurally superimposed atoms in the main GAPDH model. Overall, we observed a significant improvement of the GAPDH structure model after the MD (Supplementary Figure S1).

### GAPDH Has Four Main Regions of Contact With Interacting Proteins

We proposed 36 GAPDH complexes (Supplementary Figure S2). For each GAPDH partner, we selected two conformations with the lowest free energy scores. Supplementary Figure S2 shows GAPDH in gray, the interaction interface in dark-blue and the GAPDH partner proteins in different colors to highlight the two most stable conformations of the interaction. We chose the GAPDH interacting partners for the *in silico* validation according to the main biological function categories they participate or according to their role as regulatory proteins. There are 11 proteins related to the energy metabolism (Supplementary Figures S2A–K), three to amino acid metabolism (Supplementary Figures S2L–N), two related to virulence (Supplementary Figures S2O,P), one to fatty acid metabolism (Supplementary Figure S2Q) and one related to protein fate and complex stabilization (Supplementary Figure S2R).

**FIGURE 6:**
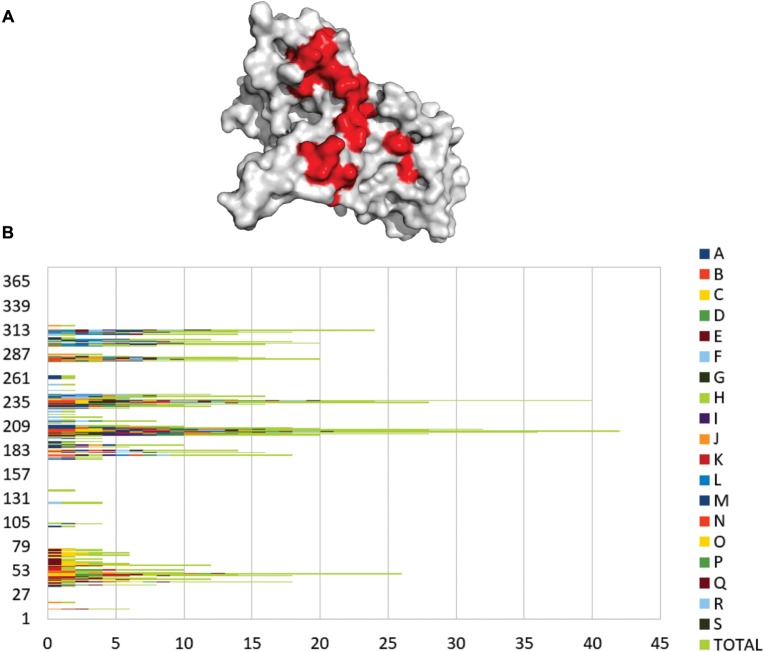
Frequent contact regions of GAPDH protein-protein interaction. **(A)** GAPDH (gray) and its four more frequent contact regions (red) of interaction with partner proteins. **(B)** The graphic shows the frequency of interaction for *18* GAPDH protein partners. Each letter and color represents a different protein that interacted with GAPDH *in silico* and the green bars represent the overall of the interactions in each residue.

Molecular docking provided the prediction of ligand orientation and possible conformations of the interacting proteins. There are at least two steps to carry out the molecular docking process. Firstly, a conformational search of the ligand proteins within a well-defined grid box around the interacting surface of GAPDH to represent different possible conformations. Then, scores of each conformation are checked and the first two scores with the lowest free energy were selected. KFC-2 server analyzes the residues within the interaction interface between GAPDH and an interacting protein to identify the contact residues. We found that four regions define the main PPIs among all the 36 analyzed complexes (Figure 6A) more frequently. The areas represented in red are the regions that contain GAPDH residues that interact with a protein partner more frequently. The identified interactions may occur *in vivo* provided GAPDH is found in the same subcellular location of its interacting partners and most of them present nitrosylation signal. The prediction of protein interacting sites has been performed successfully by several similar approaches (Aumentado-Armstrong et al., 2015; Laine and Carbone, 2015; Maheshwari and Brylinski, 2015).

Figure 6B represents an overview of the contact preference regions for all of the 18 interacting proteins selected for the present *in silico* study. The graphic shows that the GAPDH interacting region near residue 202 has the most frequent hits of interaction followed by the region 239 and 313. A large-scale study analyzed the contact preferences in 621 protein-protein complexes. They found out that those residues in the binding PPI sites are governed by hydrophobic contacts that help to maintain a stable complex conformational structure (Glaser et al., 2001). The contact preference residues on GAPDH PPI interface establish hydrophobic interactions more frequently but there are also non-polar and polar interactions involved.

## Conclusion

Here, we showed an overview of experimental GAPDH interactome in different phases of *P. lutzii*. Several proteins bound to GAPDH from mycelium (Figure 2), transition (Figure 4) and yeast (Figure 5) phases are common to important pathways such as glycolysis and TCA. We performed affinity chromatography and BN-PAGE in order to identify GAPDH partners. We found 207 proteins interacting with GAPDH, which reflects its broad spectrum of functionality and its characterization as a moonlight protein. We performed a co-immunoprecipitation assay to validate the complex formed by GAPDH with TPI, ENO, ICL and MCS. The former has a probable role in substrate channeling, the others were found in both pull down and BN-PAGE approaches. We found GAPDH participating in complexes with proteins of specific pathways, indicating the existence of a glycolytic and a TCA metabolon in *P. lutzii*. GAPDH interacted with several proteins that undergoes regulation by nitrosylation, indicating that this proteins in *P. lutzii* is involved in several metabolic process such as sensitivity to ATP intracellular levels, stabilization of multiprotein complex conformation with HSPs and regulation of mitochondrial and nuclear proteins. Moreover, *in silico* analysis showed that GAPDH has four main regions of interaction with other proteins, reflecting its conservation across evolution. Identifying GAPDH interacting proteins may shed light on the main regions of this moonlight protein that interacts with other protein partners. Finding non-conserved regions to *Homo sapiens* GAPDH can drive the search for new therapeutic approaches against PCM. Our next step is to perform the screening of new inhibiting compounds and design small molecules (peptides) to modulate pathogen GAPDH.

## Data Availability

The datasets generated for this study can be found in the ProteomeXchange Consortium via the PRIDE, PXD008253, PXD008308, PXD008309, and PXD008317.

## Author Contributions

KS contributed to the chomatography, BN-PAGE, proteomics, *in silico* analysis, and the writing of the manuscript. RL contributed to the *in silico* analysis and the writing of the manuscript. LB and PL contributed to the proteomics and writing of the manuscript. TC and SC contributed to the BN-PAGE and writing of the manuscript. RS contributed to the *in silico* analysis and writing of the manuscript. CS contributed to the writing and editing of the manuscript. MP contributed to the experimental procedures, and the writing and revision of the manuscript.

## Conflict of Interest Statement

The authors declare that the research was conducted in the absence of any commercial or financial relationships that could be construed as a potential conflict of interest.

## Footnotes

1 http://string.embl.de

2 http://www.broadinstitute.org/annotation/genome/paracoccidioides_brasiliensis/Multiome.html

3 http://www.uniprot.org/uniprot/?query=macrophage&sort=score

4 http://www.uniprot.org

5 http://mips.helmholtz-muenchen.de/funcatDB/

6 https://www.ncbi.nlm.nih.gov/

7 http://zhanglab.ccmb.med.umich.edu/I-TASSER/

8 http://molprobity.biochem.duke.edu/

9 http://vakser.compbio.ku.edu/resources/gramm/grammx/

10 https://mitchell-lab.biochem.wisc.edu/KFC_Server/index.php

11 https://www.molnac.unisa.it/BioTools/cocomaps/

## Supplementary Material

FIGURE S1 Molecular Dynamics result evaluation. Ramachandran plots showing the analysis of residues in allowed, favored and forbidden regions **(A)** before and **(B)** after GAPDH molecular dynamics. The light blue regions are favorable and the dark blue regions are allowed. Regions outside blue lines are forbidden regions and may characterize unconserved regions of GAPDH. **(C)** RMSD trajectories over 100 ns of GAPDH MD simulation. **(D)** The cluster plot shows seven sets of conformations along the GAPDH MD trajectories and the first cluster shows the most stable conformation.

FIGURE S2 Schematic representation of GAPDH interaction with 18 protein partners. Each image shows two conformations of GAPDH (gray), its protein partner (different colors) and the interacting interface (dark-blue). **(A)** malate dehydrogenase; **(B)** succinate dehydrogenase; **(C)** triosephosphate isomerase; **(D)** phosphoglycerate kinase; **(E)** cytochrome c peroxidase; **(F)** 2-methylcitrate synthase; **(G)** 2-methylcitrate dehydratase; **(H)** aconitase; **(I)** pyruvate kinase; **(J)** isocitrate lyase; **(K)** enolase; **(L)** L-threonine 3-dehydrogenase; **(M)** ribose- phosphate pyrophosphokinase; **(N)** fumarylacetoacetase; **(O)** alcohol dehydrogenase; **(P)** aldehyde dehydrogenase; **(Q)** enoyl-CoA hydratase; **(R)** HSP70.

TABLE S1 Potential GAPDH target proteins identified in *P. lutzii* mycelium through a pull down assay.

TABLE S2 Potential GAPDH target proteins identified in *P. lutzii* mycelium phase through BN-PAGE.

TABLE S3 Potential GAPDH target proteins identified in *P. lutzii* mycelium to yeast transition through a pull down assay.

TABLE S4 Potential GAPDH target proteins identified in *P. lutzii* mycelium to yeast transition through BN-PAGE.

TABLE S5 Potential GAPDH target proteins identified in *P. lutzii* yeast phase through a pull down assay.

TABLE S6 Potential GAPDH target proteins identified in *P. lutzii* yeast phase through BN-PAGE.

TABLE S7 Prediction of potential GAPDH target proteins with S-nitrosylation sites in *P. lutzii*.
