# Supplementary figures and images for: Interactome of Glyceraldehyde-3-Phosphate Dehydrogenase Points to the Existence of Metabolons in Paracoccidioides lutzii
Source: Front Microbiol. 2019 Jul 9;10:1537. doi: 10.3389/fmicb.2019.01537 (PMC6629890; doi:10.3389/fmicb.2019.01537)

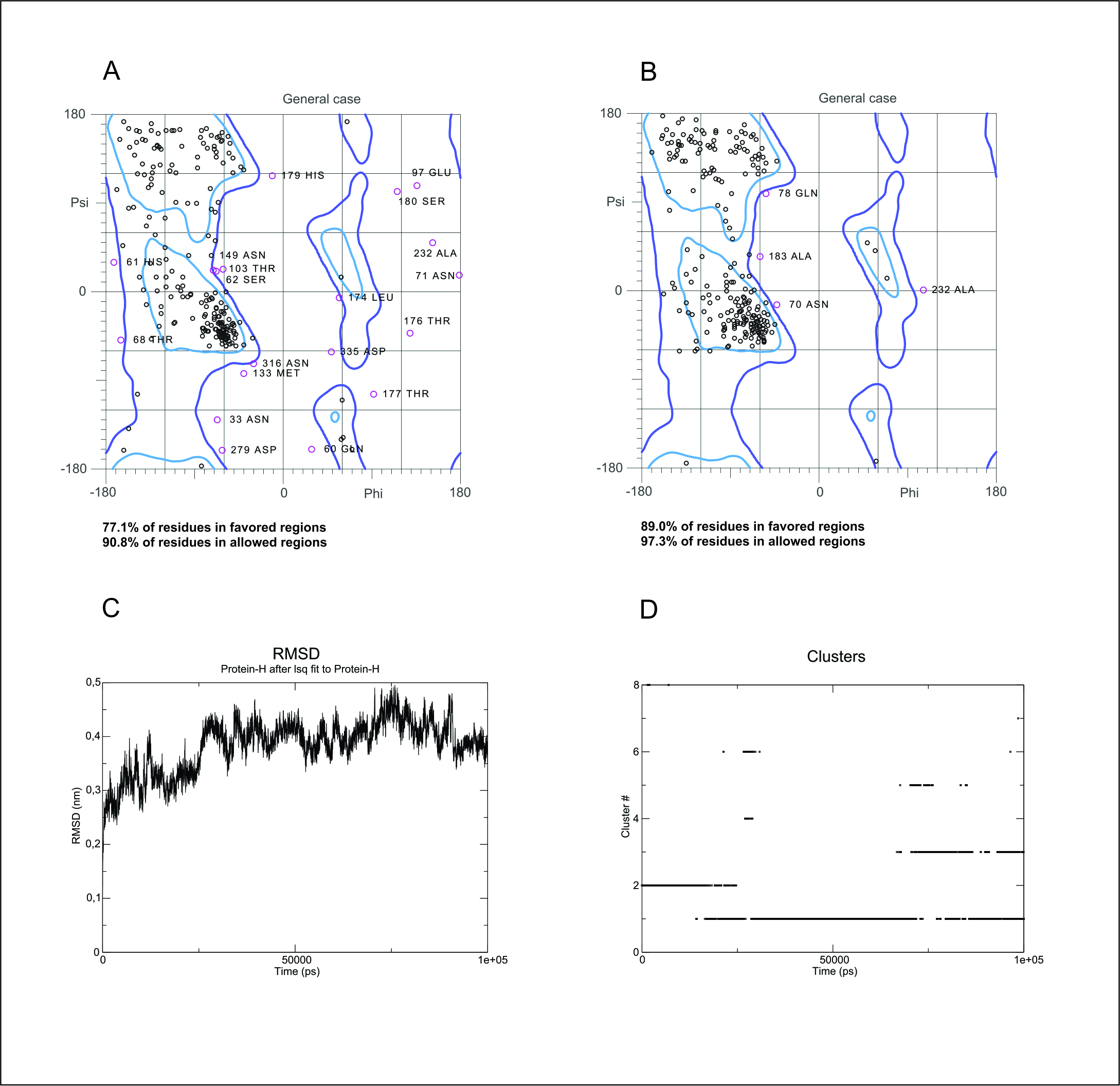

Supplement: FIGURE S1 — Molecular Dynamics result evaluation. Ramachandran plots showing the analysis of residues in allowed, favored and forbidden regions (A) before and (B) after GAPDH molecular dynamics. The light blue regions are favorable and the dark blue regions are allowed. Regions outside blue lines are forbidden regions and may characterize unconserved regions of GAPDH. (C) RMSD trajectories over 100 ns of GAPDH MD simulation. (D) The cluster plot shows seven sets of conformations along the GAPDH MD trajectories and the first cluster shows the most stable conformation. [file Image_1.JPEG]

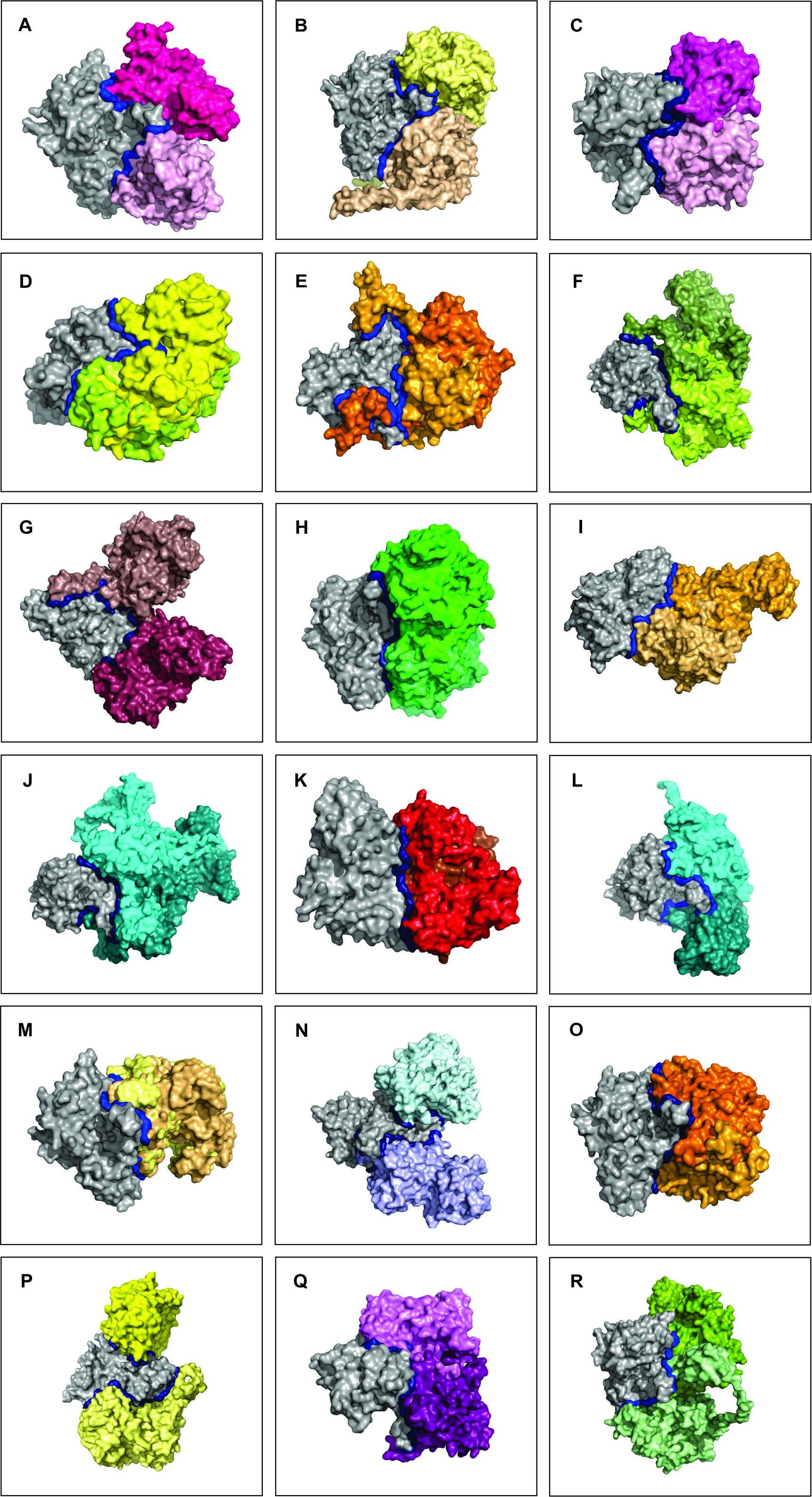

Supplement: FIGURE S2 — Schematic representation of GAPDH interaction with 18 protein partners. Each image shows two conformations of GAPDH (gray), its protein partner (different colors) and the interacting interface (dark-blue). (A) malate dehydrogenase; (B) succinate dehydrogenase; (C) triosephosphate isomerase; (D) phosphoglycerate kinase; (E) cytochrome c peroxidase; (F) 2-methylcitrate synthase; (G) 2-methylcitrate dehydratase; (H) aconitase; (I) pyruvate kinase; (J) isocitrate lyase; (K) enolase; (L) L-threonine 3-dehydrogenase; (M) ribose- phosphate pyrophosphokinase; (N) fumarylacetoacetase; (O) alcohol dehydrogenase; (P) aldehyde dehydrogenase; (Q) enoyl-CoA hydratase; (R) HSP70. [file Image_2.JPEG]
