# Supplementary material for: Interactome of Glyceraldehyde-3-Phosphate Dehydrogenase Points to the Existence of Metabolons in Paracoccidioides lutzii
Source: Front Microbiol. 2019 Jul 9;10:1537. doi: 10.3389/fmicb.2019.01537 (PMC6629890; doi:10.3389/fmicb.2019.01537)
Supplement: TABLE S1 — Potential GAPDH target proteins identified in P. lutzii mycelium through a pull down assay. [file Table_1.DOCX]

**Table 1** **-** Potential GAPDH target proteins identified in *P. lutzii* mycelium through a pull down assay

| **Acession number** | **Protein**^1^ | | **Score**^a^ |  |  |
| --- | --- | --- | --- | --- | --- |
| **1. Metabolism** |  | |  |  |  |
| **1.1 Amino acid metabolism** |  | |  |  |  |
| PAAG_07626 | cobalamin-independent synthase | | 290,5637 |  |  |
| PAAG_02859 | adenosylhomocysteinase | | 257,0193 |  |  |
| PAAG_00966 | L-threonine 3-dehydrogenase | | 315,3061 |  |  |
| PAAG_06996 | G-protein complex β subunit CpcB | | 620,7277 |  |  |
| **1.2 C-compound and carbohydrate metabolism** |  | |  |  |  |
| PAAG_12288 | UDP-N-acetylglucosamine diphosphorylase | | 395,0508 |  |  |
| **2. Energy** |  | |  |  |  |
| **2.1 Glycolysis** |  | |  |  |  |
| PAAG_11169 | enolase | | 354,8788 |  |  |
| **2.2 Glyoxylate cycle** |  | |  |  |  |
| PAAG_06951 | isocitrate lyase | | 954,309 |  |  |
| **2.3 Methylcitrate cycle** |  | |  |  |  |
| PAAG_04550 | 2-methylcitrate synthase | | 231,4709 |  |  |
| **2.4 Tricarboxylic-acid pathway** |  | |  |  |  |
| PAAG_08075 | citrate synthase | | 224,6872 |  |  |
| **2.5 Respiration** |  | |  |  |  |
| PAAG_12076 | NAD(P)H:quinone oxidoreductase | | 381,5665 |  |  |
| **3. Protein synthesis** |  | |  |  |  |
| **3.1 Ribosome biogenesis** |  | |  |  |  |
| PAAG_00430 | 60S ribosomal protein L2 | | 300,19 |  |  |
| **3.2 Translation** |  | |  |  |  |
| PAAG_04571 | nascent polypeptide-associated complex | | 633,6551 |  |  |
| **4. Protein fate** |  | |  |  |  |
| **4.1 Protein modification** |  | |  |  |  |
| PAAG_01929 | HNRNP arginine N-methyltransferase | | 406,9214 |  |  |
| **5. Cell rescue, defense and virulence** |  | |  |  |  |
| **5.1 stress response** |  | |  |  |  |
| PAAG_01454 | catalase | | 462,8666 |  |  |
| **6. Biogenesis of cellular components** |  | |  |  |  |
| **6.1 Peroxisome** |  | |  |  |  |
| PAAG_02064 | peroxin-19 (peroxisome biogenesis factor) | | 472,8071 |  |  |
| **7. Hypothetical proteins** |  | |  |  |  |
| PAAG_06562 | hypothetical protein | | 328,8647 |  |  |
| PAAG_11177 | hypothetical protein | | 640,3243 |  |  |
| PAAG_11459 | hypothetical protein | | 272,4707 |  |  |
| ^1^ Functional classification by FunCat2 (http://pedant.gsf.de/pedant3htmlview/pedant3view?Method=analysis&Db=p3_r48325_Par_lutzi) | | | | | |
| ^a^ Score: probability obtained from the Mascot search. | | |  |  |  |
