# Supplementary material for: Interactome of Glyceraldehyde-3-Phosphate Dehydrogenase Points to the Existence of Metabolons in Paracoccidioides lutzii
Source: Front Microbiol. 2019 Jul 9;10:1537. doi: 10.3389/fmicb.2019.01537 (PMC6629890; doi:10.3389/fmicb.2019.01537)
Supplement: TABLE S2 — Potential GAPDH target proteins identified in P. lutzii mycelium phase through BN-PAGE. [file Table_2.DOCX]

**Table 2** **-** Potential GAPDH target proteins identified in *P. lutzii* mycelium phase through BN-PAGE

| **Acession number** | **Protein**^1^ | | **Score**^a^ |  |  |
| --- | --- | --- | --- | --- | --- |
| **1. Metabolism** |  | |  |  |  |
| **1.1 Amino acid metabolism** |  | |  |  |  |
| PAAG_08163 | fumarylacetoacetase | | 87,9389 |  |  |
| **1.2 Purine metabolism** |  | |  |  |  |
| PAAG_04291 | nucleoside diphosphate kinase | | 187,2097 |  |  |
| **2. Energy** |  | |  |  |  |
| **2.1 Glycolysis** |  | |  |  |  |
| PAAG_11169 | enolase | | 161,465 |  |  |
| PAAG_08468 | glyceraldehyde-3-phosphate dehydrogenase | | 341,6843 |  |  |
| **2.2 Glyoxylate cycle** |  | |  |  |  |
| PAAG_06951 | isocitrate lyase | | 1340,806 |  |  |
| **2.3 Methylcitrate cycle** |  | |  |  |  |
| PAAG_04559 | 2-methylcitrate dehydratase | | 102,7028 |  |  |
| **2.4 Tricarboxylic-acid pathway** |  | |  |  |  |
| PAAG_05048 | aconitase | | 107,0994 |  |  |
| **3. Protein synthesis** |  | |  |  |  |
| **3.1 Translation elongation** |  | |  |  |  |
| PAAG_00594 | elongation factor 2 | | 270,8449 |  |  |
| **4. Protein fate** |  | |  |  |  |
| **4.1 Protein folding and stabilization** |  | |  |  |  |
| PAAG_11262 | hsp7 | | 252,9421 |  |  |
| PAAG_05142 | hsp10 | | 523,5358 |  |  |
| PAAG_08059 | hsp60 | | 163,5902 |  |  |
| PAAG_08003 | hsp70 | | 306,3665 |  |  |
| PAAG_01262 | hsp70 | | 216,5211 |  |  |
| PAAG_07750 | hsp88 | | 160,9349 |  |  |
| PAAG_05679 | hsp90 | | 113,4504 |  |  |
| ^1^ Functional classification by FunCat2 (http://pedant.gsf.de/pedant3htmlview/pedant3view?Method=analysis&Db=p3_r48325_Par_lutzi) | | | | | |
| ^a^ Score: probability obtained from the Mascot search. | | |  |  |  |
