# Supplementary material for: Interactome of Glyceraldehyde-3-Phosphate Dehydrogenase Points to the Existence of Metabolons in Paracoccidioides lutzii
Source: Front Microbiol. 2019 Jul 9;10:1537. doi: 10.3389/fmicb.2019.01537 (PMC6629890; doi:10.3389/fmicb.2019.01537)
Supplement: TABLE S3 — Potential GAPDH target proteins identified in P. lutzii mycelium to yeast transition through a pull down assay. [file Table_3.DOCX]

**Table 3** **-** Potential GAPDH target proteins identified in *P. lutzii* mycelium to yeast transition through a pull down assay

| **Acession number** | **Protein**^1^ | **Score**^a^ | |  |  |
| --- | --- | --- | --- | --- | --- |
| **1. Metabolism** |  |  | |  |  |
| **1.1 Amino acid metabolism** |  |  | |  |  |
| PAAG_04701 | cystathionine γ-lyase | 758,2913 | |  |  |
| PAAG_08512 | serine hydroxymethyltransferase | 755,6329 | |  |  |
| PAAG_04102 | isovaleryl-CoA dehydrogenase | 1099,97 | |  |  |
| PAAG_04103 | methylcrotonoyl-CoA carboxylase β chain | 1284,13 | |  |  |
| PAAG_04205 | vacuolar aminopeptidase | 1356,54 | |  |  |
| PAAG_01321 | oxidoreductase 2-nitropropane dioxygenase family | 2127,19 | |  |  |
| PAAG_00966 | L-threonine 3-dehydrogenase | 912,497 | |  |  |
| **1.2 Nucleotide/nucleoside/nucleobase metabolism** | |  | |  |  |
| PAAG_05611 | deoxyuridine 5'-triphosphate nucleotidohydrolase | 2648,69 | |  |  |
| PAAG_11311 | pyridine-specific 5'-nucleotidase | 952,812 | |  |  |
| **1.3 C-compound and carbohydrate metabolism** | |  | |  |  |
| PAAG_06817 | UTP-glucose-1-phosphate uridylyltransferase | 6000,54 | |  |  |
| **1.4 Fatty acid metabolism** |  |  | |  |  |
| PAAG_00435 | (R)-benzylsuccinyl-CoA dehydrogenase | 1253,3 | |  |  |
| **2. Energy** |  |  | |  |  |
| **2.1 Tricarboxylic-acid pathway** | |  | |  |  |
| PAAG_08449 | malate dehydrogenase* | 3556,86 | |  |  |
| **2.2 Respiration** |  |  | |  |  |
| PAAG_08037 | ATP synthase subunit β* | 1266,38 | |  |  |
| PAAG_12076 | NAD(P)H:quinone oxidoreductase | 1731,47 | |  |  |
| **3. Cell cycle and DNA processing** | |  | |  |  |
| **3.1 Cell cycle** |  |  | |  |  |
| PAAG_00182 | cell division control protein | 686,785 | |  |  |
| PAAG_08880 | cell division control protein | 1731,43 | |  |  |
| PAAG_08917 | histone H2A | 8445,98 | |  |  |
| PAAG_01647 | tubulin α-1 chain | 1400,19 | |  |  |
| PAAG_01347 | actin cytoskeleton protein (VIP1) | 1834,8 | |  |  |
| PAAG_05863 | M protein repeat protein | 1121,3 | |  |  |
| **4. Transcription** |  |  | |  |  |
| **4.1 RNA synthesis** |  |  | |  |  |
| PAAG_07204 | pH-response transcription factor pacC/RIM101 | 1117,99 | |  |  |
| **4.2 RNA processing** |  |  | |  |  |
| PAAG_00101 | small nuclear ribonucleoprotein | 4241,89 | |  |  |
| PAAG_02522 | U6 snRNA-associated Sm-like protein LSm3 | 9647,9 | |  |  |
| PAAG_12408 | U6 snRNA-associated Sm-like protein LSm8 | 5669,01 | |  |  |
| **5. Protein synthesis** |  |  | |  |  |
| **5.1 Ribosome biogenesis** |  |  | |  |  |
| PAAG_09043 | 40S ribosomal protein S2 | 1196,87 | |  |  |
| PAAG_05484 | 40S ribosomal protein S5 | 1035,44 | |  |  |
| PAAG_04690 | 40S ribosomal protein S15 | 4801,94 | |  |  |
| PAAG_09096 | 40S ribosomal protein S28 | 2573,69 | |  |  |
| PAAG_08888 | 60S ribosomal protein L4 | 1038,42 | |  |  |
| PAAG_00347 | 60S ribosomal protein L9 | 3633,73 | |  |  |
| PAAG_07385 | 60S ribosomal protein L25 | 3633,73 | |  |  |
| PAAG_08847 | 60S ribosomal protein L28 | 2444,62 | |  |  |
| PAAG_07841 | 60S acidic ribosomal protein P1 | 8146,49 | |  |  |
| PAAG_04651 | GTP-binding nuclear protein GSP1/Ran | 1956,88 | |  |  |
| PAAG_02940 | TIA1 cytotoxic granule-associated RNA binding protein | 758,471 | |  |  |
| **5.2 Translation** |  |  | |  |  |
| PAAG_00689 | ATP-dependent RNA helicase eIF4A | 1968,47 | |  |  |
| PAAG_00594 | elongation factor 2 | 1006,37 | |  |  |
| PAAG_12263 | translation initiation factor 3 subunit M | 2251,08 | |  |  |
| PAAG_00376 | eukaryotic translation initiation factor 3 subunit F | 1183,49 | |  |  |
| PAAG_01425 | eukaryotic translation initiation factor 3 | 928,058 | |  |  |
| PAAG_11075 | eukaryotic translation initiation factor 5A | 840,37 | |  |  |
| **6. Protein fate** |  |  | |  |  |
| **6.1 Protein folding and stabilization** | |  | |  |  |
| PAAG_06255 | hsp24 | 1846,12 | |  |  |
| PAAG_08003 | hsp70* | 3837,37 | |  |  |
| PAAG_01262 | hsp70* | 969,183 | |  |  |
| PAAG_05679 | hsp90* | 2582,9 | |  |  |
| **6.2 Stress Response** | |  | |  |  |
| PAAG_02364 | thioredoxin | 2220,51 | |  |  |
| **7. Hypothetical proteins** |  |  | |  |  |
| PAAG_00365 | hypothetical protein | 2788,75 | |  |  |
| PAAG_01112 | hypothetical protein | 1122,64 | |  |  |
| PAAG_03129 | hypothetical protein | 2816,91 | |  |  |
| PAAG_04647 | hypothetical protein | 15661,5 | |  |  |
| PAAG_05699 | hypothetical protein | 912,268 | |  |  |
| PAAG_07692 | hypothetical protein | 1286,84 | |  |  |
| PAAG_07792 | hypothetical protein | 1436,24 | |  |  |
| PAAG_07830 | hypothetical protein | 1388,37 | |  |  |
| ^1^ Functional classification by FunCat2 (http://pedant.gsf.de/pedant3htmlview/pedant3view?Method=analysis&Db=p3_r48325_Par_lutzi) | | | | | |
| ^a^ Score: probability obtained from the Mascot search.  * Proteins bound to GAPDH during mycelium-to-yeast transition that were up-regulated in this same phase in *P. brasiliensis* (Rezende et al., 2011). | | | |  |  |
