# Supplementary material for: Interactome of Glyceraldehyde-3-Phosphate Dehydrogenase Points to the Existence of Metabolons in Paracoccidioides lutzii
Source: Front Microbiol. 2019 Jul 9;10:1537. doi: 10.3389/fmicb.2019.01537 (PMC6629890; doi:10.3389/fmicb.2019.01537)
Supplement: TABLE S4 — Potential GAPDH target proteins identified in P. lutzii mycelium to yeast transition through BN-PAGE. [file Table_4.DOCX]

**Table 4** **-** Potential GAPDH target proteins identified in *P. lutzii* mycelium to yeast transition through BN-PAGE

| **Acession number** | **Protein**^1^ | | **Score**^a^ |  |  |
| --- | --- | --- | --- | --- | --- |
| **1. Metabolism** |  | |  |  |  |
| **1.1 Amino acid metabolism** |  | |  |  |  |
| PAAG_02859 | adenosylhomocysteinase* | | 123,5245 |  |  |
| **1.5 C-compound and carbohydrate metabolism** |  | |  |  |  |
| PAAG_06473 | mannitol-1-phosphate 5-dehydrogenase | | 197,4853 |  |  |
| PAAG_04541 | alcohol dehydrogenase* | | 156,227 |  |  |
| PAAG_05249 | aldehyde dehydrogenase | | 134,6697 |  |  |
| **1.6 Fatty acid metabolism** |  | |  |  |  |
| PAAG_06309 | enoyl-CoA hydratase | | 54,9421 |  |  |
| PAAG_06329 | 3-hydroxybutyryl-CoA dehydrogenase | | 359,9208 |  |  |
| **2. Energy** |  | |  |  |  |
| **2.1 Glycolysis** |  | |  |  |  |
| PAAG_02585 | triosephosphate isomerase | | 836,5897 |  |  |
| PAAG_08468 | glyceraldehyde-3-phosphate dehydrogenase | | 136,2865 |  |  |
| **2.2 Respiration** |  | |  |  |  |
| PAAG_12076 | NAD(P)H:quinone oxidoreductase | | 320,6653 |  |  |
| **3. Cell cycle and DNA processing** |  | |  |  |  |
| **3.1 DNA processing** |  | |  |  |  |
| PAAG_00923 | proliferating cell nuclear antigen | | 122,4656 |  |  |
| **3.2 cell cycle** |  | |  |  |  |
| PAAG_00773 | 14-3-3 protein | | 1182,651 |  |  |
| PAAG_06751 | DNA damage checkpoint protein rad24 | | 1048,402 |  |  |
| PAAG_08358 | FAD-linked sulfhydryl oxidase ALR | | 132,6738 |  |  |
| **4. Protein fate** |  | |  |  |  |
| **4.1 Protein folding and stabilization** |  | |  |  |  |
| PAAG_08003 | hsp70* | | 28,5555 |  |  |
| PAAG_00986 | disulfide isomerase Pdi1 | | 671,9004 |  |  |
| PAAG_03334 | peptidyl-prolyl cis-trans isomerase D | | 683,9595 |  |  |
| **Hypothetical proteins** |  | |  |  |  |
| PAAG_11312 | hypothetical protein | | 519,9568 |  |  |
| PAAG_06036 | hypothetical protein | | 72,314 |  |  |
| ^1^ Functional classification by FunCat2 (http://pedant.gsf.de/pedant3htmlview/pedant3view?Method=analysis&Db=p3_r48325_Par_lutzi) | | | | | |
| ^a^ Score: probability obtained from the Mascot search. | | |  |  |  |

* Proteins bound to GAPDH during mycelium-to-yeast transition that were up-regulated in this same phase in *P. brasiliensis* (Rezende et al., 2011).
