# Supplementary material for: Interactome of Glyceraldehyde-3-Phosphate Dehydrogenase Points to the Existence of Metabolons in Paracoccidioides lutzii
Source: Front Microbiol. 2019 Jul 9;10:1537. doi: 10.3389/fmicb.2019.01537 (PMC6629890; doi:10.3389/fmicb.2019.01537)
Supplement: TABLE S5 — Potential GAPDH target proteins identified in P. lutzii yeast phase through a pull down assay. [file Table_5.DOCX]

**Table 5** **-** Potential GAPDH target proteins identified in *P. lutzii* yeast phase through a pull down assay

| **Acession number** | **Protein**^1^ | | **Score**^a^ |  |  |
| --- | --- | --- | --- | --- | --- |
| **1. Metabolism** |  | |  |  |  |
| **1.1 Amino acid metabolism** |  | |  |  |  |
| PAAG_08100 | *O*-acetylhomoserine (thiol)-lyase* | | 2194,755 |  |  |
| PAAG_04701 | cystathionine γ-lyase | | 1161,111 |  |  |
| PAAG_02603 | aspartate aminotransferase | | 723,5638 |  |  |
| PAAG_02115 | ribose-phosphate pyrophosphokinase | | 781,5906 |  |  |
| PAAG_07114 | argininosuccinate synthase | | 696,4496 |  |  |
| PAAG_07626 | cobalamin-independent synthase* | | 1064,227 |  |  |
| PAAG_08065 | aspartate-semialdehyde dehydrogenase | | 882,6921 |  |  |
| PAAG_08164 | homogentisate 1,2-dioxygenase | | 783,8858 |  |  |
| PAAG_04102 | isovaleryl-CoA dehydrogenase | | 47248,81 |  |  |
| PAAG_02859 | adenosylhomocysteinase | | 1632,847 |  |  |
| PAAG_01321 | oxidoreductase 2-nitropropane dioxygenase* | | 3414,967 |  |  |
| PAAG_06237 | urease accessory protein ureG | | 6952,267 |  |  |
| PAAG_00966 | L-threonine 3-dehydrogenase* | | 2551,646 |  |  |
| **1.2 Nucleotide/nucleoside/nucleobase metabolism** | | |  |  |  |
| PAAG_00731 | bifunctional purine biosynthesis protein ADE17 | | 1957,853 |  |  |
| PAAG_04291 | nucleoside diphosphate kinase* | | 2705,946 |  |  |
| **1.3 C-compound and carbohydrate metabolism** | | |  |  |  |
| PAAG_00545 | glycogen phosphorylase | | 699,6829 |  |  |
| **1.4 Fatty acid metabolism** |  | |  |  |  |
| PAAG_00435 | (R)-benzylsuccinyl-CoA dehydrogenase | | 1441,405 |  |  |
| PAAG_02664 | 3-ketoacyl-CoA thiolase | | 1126,571 |  |  |
| PAAG_07786 | acetyl-CoA acetyltransferase | | 1068,865 |  |  |
| PAAG_05454 | acyl-CoA dehydrogenase | | 910,3766 |  |  |
| **2. Energy** |  | |  |  |  |
| **2.1 Glycolysis and gluconeogenese** |  | |  |  |  |
| PAAG_02585 | triosephosphate isomerase* | | 1230,095 |  |  |
| PAAG_02869 | phosphoglycerate kinase* | | 1256,681 |  |  |
| PAAG_06380 | pyruvate kinase | | 970,5694 |  |  |
| PAAG_02050 | pyruvate decarboxylase | | 4327,15 |  |  |
| **2.2 Glyoxylate cycle** |  | |  |  |  |
| PAAG_06951 | isocitrate lyase* | | 801,7766 |  |  |
| **2.3 Methylcitrate cycle** |  | |  |  |  |
| PAAG_04550 | 2-methylcitrate synthase | | 7269,288 |  |  |
| PAAG_04559 | 2-methylcitrate dehydratase* | | 1140,824 |  |  |
| **2.4 Tricarboxylic-acid pathway** |  | |  |  |  |
| PAAG_00856 | isocitrate dehydrogenase | | 979,7418 |  |  |
| PAAG_01725 | succinate dehydrogenase flavoprotein subunit | | 692,6098 |  |  |
| PAAG_00053 | malate dehydrogenase | | 4558,244 |  |  |
| PAAG_01534 | pyruvate dehydrogenase E1 component subunit β | | 5164,968 |  |  |
| PAAG_03330 | dihydrolipoyl dehydrogenase | | 856,7659 |  |  |
| PAAG_08075 | citrate synthase | | 1017,464 |  |  |
| **2.5 Respiration** |  | |  |  |  |
| PAAG_08037 | ATP synthase subunit β | | 6840,705 |  |  |
| PAAG_03292 | cytochrome c peroxidase | | 903,4189 |  |  |
| PAAG_12076 | NAD(P)H:quinone oxidoreductase_ type IV | | 2120,031 |  |  |
| **3. Cell cycle and DNA processing** |  | |  |  |  |
| **3.1 Cell cycle** |  | |  |  |  |
| PAAG_00773 | 14-3-3 protein | | 1387,298 |  |  |
| PAAG_01647 | tubulin α-1 chain | | 942,1626 |  |  |
| PAAG_08917 | histone H2A | | 11238,75 |  |  |
| PAAG_07098 | histone H4.1 | | 4084,716 |  |  |
| PAAG_00126 | histone H4.2 | | 4823,113 |  |  |
| PAAG_05518 | cell division cycle protein | | 5233,908 |  |  |
| PAAG_03532 | actin | | 6199,279 |  |  |
| **4. Protein synthesis** |  | |  |  |  |
| **4.1 Ribosome biogenesis** |  | |  |  |  |
| PAAG_08955 | 40S ribosomal protein S1 | | 13011,11 |  |  |
| PAAG_09043 | 40S ribosomal protein S2 | | 5749,854 |  |  |
| PAAG_03816 | 40S ribosomal protein S4 | | 1372,313 |  |  |
| PAAG_05484 | 40S ribosomal protein S5 | | 8714,349 |  |  |
| PAAG_02634 | 40S ribosomal protein S6 | | 4948,12 |  |  |
| PAAG_07182 | 40S ribosomal protein S7 | | 2863,506 |  |  |
| PAAG_03828 | 40S ribosomal protein S9 | | 2646,603 |  |  |
| PAAG_06367 | 40S ribosomal protein S11 | | 4586,104 |  |  |
| PAAG_08634 | 40S ribosomal protein S12 | | 3039,11 |  |  |
| PAAG_05704 | 40S ribosomal protein S13 | | 956,1746 |  |  |
| PAAG_01433 | 40S ribosomal protein S14 | | 3121,067 |  |  |
| PAAG_04690 | 40S ribosomal protein S15 | | 31331,08 |  |  |
| PAAG_01413 | 40S ribosomal protein S17 | | 1514,983 |  |  |
| PAAG_05337 | 40S ribosomal protein S22 | | 2694,15 |  |  |
| PAAG_00385 | 40S ribosomal protein S23 | | 2078,107 |  |  |
| PAAG_06882 | 40S ribosomal protein S24 | | 3593,009 |  |  |
| PAAG_09096 | 40S ribosomal protein S28 | | 7973,425 |  |  |
| PAAG_00088 | 60S ribosomal protein L3 | | 4398,236 |  |  |
| PAAG_11125 | 60S ribosomal protein L5 | | 861,6821 |  |  |
| PAAG_06487 | 60S ribosomal protein L7 | | 3901,373 |  |  |
| PAAG_04998 | 60S ribosomal protein L8 | | 1617,961 |  |  |
| PAAG_01052 | 60S ribosomal protein L10 | | 2782,447 |  |  |
| PAAG_00724 | 60S ribosomal protein L11 | | 2952,298 |  |  |
| PAAG_05379 | 60S ribosomal protein L17 | | 2070,573 |  |  |
| PAAG_07955 | 60S ribosomal protein L18 | | 6772,223 |  |  |
| PAAG_07385 | 60S ribosomal protein L25 | | 7324,559 |  |  |
| PAAG_00801 | 60S acidic ribosomal protein P0 | | 1049,871 |  |  |
| PAAG_07841 | 60S acidic ribosomal protein P1 | | 3599,521 |  |  |
| PAAG_09083 | TCTP family protein | | 7137,488 |  |  |
| PAAG_04651 | GTP-binding nuclear protein GSP1/Ran | | 2214,736 |  |  |
| **4.2 Translation** |  | |  |  |  |
| PAAG_00689 | ATP-dependent RNA helicase eIF4A | | 1190,729 |  |  |
| PAAG_03028 | elongation factor 1-β* | | 2210,912 |  |  |
| PAAG_03556 | elongation factor 1-γ domain-containing protein* | | 3444,259 |  |  |
| PAAG_02921 | elongation factor Tu | | 1195,99 |  |  |
| **5. Protein fate** |  | |  |  |  |
| **5.1 Protein folding and stabilization** |  | |  |  |  |
| PAAG_03334 | peptidyl-prolyl cis-trans isomerase D* | | 3878,823 |  |  |
| PAAG_01727 | T-complex protein 1 subunit delta | | 843,9988 |  |  |
| PAAG_00986 | disulfide isomerase Pdi1 | | 1046,603 |  |  |
| PAAG_06255 | hsp24 | | 1961,501 |  |  |
| PAAG_00871 | hsp30 | | 3638,559 |  |  |
| PAAG_07444 | hsp70 | | 1112,857 |  |  |
| PAAG_07750 | hsp88 | | 2606,034 |  |  |
| PAAG_05679 | hsp90 | | 7905,668 |  |  |
| PAAG_07775 | hsp SSB1 | | 1365,927 |  |  |
| PAAG_12063 | protein transporter SEC23 | | 1002,6 |  |  |
| **6. Hypothetical proteins** |  | |  |  |  |
| PAAG_05019 | hypothetical protein | | 3227,077 |  |  |
| ^1^ Functional classification by FunCat2 (http://pedant.gsf.de/pedant3htmlview/pedant3view?Method=analysis&Db=p3_r48325_Par_lutzi) | | | | | |
| ^a^ Score: probability obtained from the Mascot search. | | |  |  |  |

* Proteins bound to GAPDH during yeast phase that were up-regulated in this same phase in *P. brasiliensis*.
