# Supplementary material for: Interactome of Glyceraldehyde-3-Phosphate Dehydrogenase Points to the Existence of Metabolons in Paracoccidioides lutzii
Source: Front Microbiol. 2019 Jul 9;10:1537. doi: 10.3389/fmicb.2019.01537 (PMC6629890; doi:10.3389/fmicb.2019.01537)
Supplement: TABLE S6 — Potential GAPDH target proteins identified in P. lutzii yeast phase through BN-PAGE. [file Table_6.DOCX]

**Table 6** **-** Potential GAPDH target proteins identified in *P. lutzii* yeast phase through BN-PAGE

| **Acession number** | **Protein**^1^ | | **Score**^a^ |  |  |
| --- | --- | --- | --- | --- | --- |
| **1. Metabolism** |  | |  |  |  |
| **1.1 C-compound and carbohydrate metabolism** |  | |  |  |  |
| PAAG_05249 | aldehyde dehydrogenase | | 2011,805 |  |  |
| PAAG_04541 | alcohol dehydrogenase* | | 315,2837 |  |  |
| **1.2 Fatty acid metabolism** |  | |  |  |  |
| PAAG_03447 | acetyl-CoA acetyltransferase | | 314,8989 |  |  |
| PAAG_06309 | enoyl-CoA hydratase* | | 184,249 |  |  |
| PAAG_06329 | 3-hydroxybutyryl-CoA dehydrogenase | | 345,6584 |  |  |
| **2. Energy** |  | |  |  |  |
| **2.1 Glycolysis and gluconeogenese** |  | |  |  |  |
| PAAG_08468 | glyceraldehyde-3-phosphate dehydrogenase | | 16476,82 |  |  |
| PAAG_08203 | phosphoenolpyruvate carboxykinase | | 87,7078 |  |  |
| **2.2 Glyoxylate cycle** |  | |  |  |  |
| PAAG_06951 | isocitrate lyase* | | 634,2487 |  |  |
| **2.3 Methylcitrate cycle** |  | |  |  |  |
| PAAG_04550 | 2-methylcitrate synthase | | 215,7776 |  |  |
| PAAG_04559 | 2-methylcitrate dehydratase* | | 1000,293 |  |  |
| **2.4 Energy conservation and regeneration** |  | |  |  |  |
| PAAG_03631 | 12-oxophytodienoate reductase* | | 538,4603 |  |  |
| **3. Cell cycle and DNA processing** |  | |  |  |  |
| **3.1 DNA processing** |  | |  |  |  |
| PAAG_04609 | mis6 domain-containing protein | | 104,1578 |  |  |
| **4. Protein synthesis** |  | |  |  |  |
| **4.1 Ribosome biogenesis** |  | |  |  |  |
| PAAG_00801 | 60S acidic ribosomal protein P0 | | 66,3752 |  |  |
| PAAG_04726 | pirin | | 78,8068 |  |  |
| PAAG_02837 | eukaryotic translation initiation factor 3 subunit H | | 67,522 |  |  |
| **5. Protein fate** |  | |  |  |  |
| **5.1 Protein folding and stabilization** |  | |  |  |  |
| PAAG_11262 | hsp7 | | 178,2185 |  |  |
| PAAG_08003 | hsp70 | | 133,1478 |  |  |
| PAAG_05679 | hsp90 | | 95,8979 |  |  |
| **6. Cellular transport** |  | |  |  |  |
| **6.1 Transported compounds** |  | |  |  |  |
| PAAG_04276 | phosphatidylinositol transporter | | 103,1025 |  |  |
| **7. Signal transduction** |  | |  |  |  |
| **7.1 Cellular signaling** |  | |  |  |  |
| PAAG_01842 | β-lactamase family protein | | 66,6552 |  |  |
| **8. Hypothetical proteins** |  | |  |  |  |
| PAAG_02487 | hypothetical protein | | 158,8956 |  |  |
| PAAG_11312 | hypothetical protein | | 1899,092 |  |  |
| PAAG_03233 | hypothetical protein | | 76,0344 |  |  |
| PAAG_12093 | hypothetical protein | | 68,1047 |  |  |
| PAAG_11068 | hypothetical protein | | 90,3262 |  |  |
| PAAG_01271 | hypothetical protein | | 69,8253 |  |  |
| ^1^ Functional classification by FunCat2 (http://pedant.gsf.de/pedant3htmlview/pedant3view?Method=analysis&Db=p3_r48325_Par_lutzi) | | | | | |
| ^a^ Score: probability obtained from the Mascot search. | | |  |  |  |

* Proteins bound to GAPDH during yeast phase that were up-regulated in this same phase in *P. brasiliensis*.
