# Supplementary material for: Interactome of Glyceraldehyde-3-Phosphate Dehydrogenase Points to the Existence of Metabolons in Paracoccidioides lutzii
Source: Front Microbiol. 2019 Jul 9;10:1537. doi: 10.3389/fmicb.2019.01537 (PMC6629890; doi:10.3389/fmicb.2019.01537)
Supplement: TABLE S7 — Prediction of potential GAPDH target proteins with S-nitrosylation sites in P. lutzii. [file Table_7.DOCX]

| **Access** | **GAPDH partner** | **Position** | **Peptide** | **Score^1^** | **Cutoff^2^** | **Cluster^3^** |
| --- | --- | --- | --- | --- | --- | --- |
| PAAG_05048 | aconitase | 104 | LRPDRVACQDATAQM | 2,902 | 2,454 | B |
| PAAG_02859 | adenosylhomocysteinase | 198 | KFDNLYGCRESLVDG | 3,592 | 2,454 | B |
| PAAG_00966 | L-threonine 3-dehydrogenase | 331 | KIEQWAECLESMRNK | 2,864 | 2,454 | B |
| PAAG_11169 | ENO | 47 | STGQHEACELRDGDQ | 21,693 | 20,743 | C |
| PAAG_01929 | HNRNP arginine N-methyltransferase | 76 | KIVLDVGCGTGILSM | 3,391 | 2,454 | B |
| PAAG_01929 | HNRNP arginine N-methyltransferase | 347 | IRAAQGSCEYKM | 2,516 | 2,454 | B |
| PAAG_01454 | catalase | 24 | TYTTSNGCPVMDPES | 22,416 | 20,743 | C |
| PAAG_02064 | peroxin-19 | 420 | GPGDIPECPQQ | 2,946 | 2,454 | B |
| PAAG_08163 | fumarylacetoacetase | 418 | SYVGFGDCTAVILPA | 2,679 | 2,454 | B |
| PAAG_00594 | elongation factor 2 | 132 | GALVVVDCVSGVCVQ | 2,783 | 2,454 | B |
| PAAG_00594 | elongation factor 2 | 517 | RLSKSDPCVLTYISE | 23,343 | 20,743 | C |
| PAAG_00594 | elongation factor 2 | 541 | GELHLEICLKDLEED | 2,761 | 2,454 | B |
| PAAG_11262 | HSP 7 | 308 | IQRIREACEKAKIEL | 1,842 | 1,67 | A |
| PAAG_08003 | HSP 70 | 63 | DLGTTYSCVGAMQNG | 2,918 | 2,454 | B |
| PAAG_07750 | HSP 88 | 198 | EEKPRRVCFVDIGHS | 21,664 | 20,743 | C |
| PAAG_05679 | HSP 90 | 571 | HKLIGSPCAIRTGQF | 20,978 | 20,743 | C |
| PAAG_08512 | serine hydroxymethyltransferase | 6 | MSLNICSRRAAAR | 3,87 | 2,454 | B |
| PAAG_08512 | serine hydroxymethyltransferase | 133 | IDQAETLCQQRALKA | 2,668 | 2,454 | B |
| PAAG_04102 | isovaleryl-CoA dehydrogenase | 5 | MASPCLPRLLGR | 4,766 | 2,454 | B |
| PAAG_04102 | isovaleryl-CoA dehydrogenase | 367 | NAIRKQDCAGAILYA | 4,929 | 2,454 | B |
| PAAG_00435 | (R)-benzylsuccinyl-CoA dehydrogenase | 190 | SGAGDPRCKVYIVMG | 22,898 | 20,743 | C |
| PAAG_00435 | (R)-benzylsuccinyl-CoA dehydrogenase | 420 | NKQRRDECTEKIRRQ | 2,701 | 2,454 | B |
| PAAG_08037 | ATP synthase subunit β | 36 | ESRFESRCALFLPVS | 20,942 | 20,743 | C |
| PAAG_01647 | tubulin α-1 chain | 6 | **MRGEVCHLHIGQA | 23,219 | 20,743 | C |
| PAAG_01647 | tubulin α-1 chain | 348 | SFNLVEWCPTGFKIG | 1,803 | 1,67 | A |
| PAAG_05484 | 40S ribosomal protein S5 | 182 | NVKSIAECLAEELIN | 2,467 | 2,454 | B |
| PAAG_00347 | 60S ribosomal protein L9 | 167 | AADIQQICRVRNKDI | 21,591 | 20,743 | C |
| PAAG_08847 | 60S ribosomal protein L28 | 803 | REFYENSCMRTVNQC | 2,777 | 2,454 | B |
| PAAG_08847 | 60S ribosomal protein L28 | 810 | CMRTVNQCIGRAIRH | 2,739 | 2,454 | B |
| PAAG_04651 | GTP-binding nuclear protein GSP1/Ran | 109 | HRDLTRVCENIPIVL | 2,262 | 1,67 | A |
| PAAG_04651 | GTP-binding nuclear protein GSP1/Ran | 117 | ENIPIVLCGNKVDVK | 3,174 | 2,454 | B |
| PAAG_02940 | TIA1 cytotoxic granule-associated RNA binding protein | 318 | TPQWQTTCYVGNLTP | 20,759 | 20,743 | C |
| PAAG_02940 | TIA1 cytotoxic granule-associated RNA binding protein | 369 | ENAAMAICQLSGYNV | 21,883 | 20,743 | C |
| PAAG_00689 | ATP-dependent RNA helicase eIF4A | 398 | PMNVAGMC******* | 2,011 | 1,67 | A |
| PAAG_11075 | eukaryotic translation initiation factor 5A | 130 | SKKLEDLCPSTHNME | 2,06 | 1,67 | A |
| PAAG_01112 | hypothetical protein | 73 | FAAEIRNCAGSSVSF | 2,489 | 2,454 | B |
| PAAG_04541 | alcohol dehydrogenase | 157 | GDTVLISCITACGGC | 2,505 | 2,454 | B |
| PAAG_05249 | aldehyde dehydrogenase | 400 | EEIFGPVCCVQKFKS | 21,051 | 20,743 | C |
| PAAG_06329 | 3-hydroxybutyryl-CoA dehydrogenase | 242 | YINEAIMCLENGVGT | 2,75 | 2,454 | B |
| PAAG_02585 | triosephosphate isomerase | 125 | GGLNVILCIGESLEE | 4,571 | 2,454 | B |
| PAAG_00923 | proliferating cell nuclear antigen | 239 | KDGVKFSCQGEIGNG | 4,185 | 2,454 | B |
| PAAG_00773 | 14-3-3 protein | 15 | KTFLARLCEQAERYD | 4,087 | 1,67 | A |
| PAAG_00773 | 14-3-3 protein | 98 | ETELERVCQDVLDVL | 2,497 | 1,67 | A |
| PAAG_06751 | DNA damage checkpoint protein rad24 | 96 | ETELAKICEDILDVL | 2,519 | 1,67 | A |
| PAAG_00986 | disulfide isomerase Pdi1 | 57 | AEFYAPWCGHCKALA | 1,787 | 1,67 | A |
| PAAG_00986 | disulfide isomerase Pdi1 | 60 | YAPWCGHCKALAPEY | 1,825 | 1,67 | A |
| PAAG_00986 | disulfide isomerase Pdi1 | 392 | LEFYAPWCGHCKALA | 1,765 | 1,67 | A |
| PAAG_00986 | disulfide isomerase Pdi1 | 395 | YAPWCGHCKALAPKY | 2,399 | 1,67 | A |
| PAAG_06036 | hypothetical protein | 17 | SRALCMRCLIVGEVP | 22,365 | 20,743 | C |
| PAAG_07114 | argininosuccinate synthase | 351 | NGQVRLRCYKGLTSV | 2,75 | 2,454 | B |
| PAAG_08065 | aspartate-semialdehyde dehydrogenase | 9 | STTTKKRCGVLGATG | 23,204 | 20,743 | C |
| PAAG_00731 | bifunctional purine biosynthesis protein ADE17 | 170 | RAAAKNHCRVTILSD | 22,066 | 20,743 | C |
| PAAG_02664 | 3-ketoacyl-CoA thiolase | 117 | VKSLNRQCSSSLQAI | 1,71 | 1,67 | A |
| PAAG_02664 | 3-ketoacyl-CoA thiolase | 172 | NNQEAANCKVPMGVL | 22,613 | 20,743 | C |
| PAAG_02869 | phosphoglycerate kinase | 99 | SVVFTDDCVGKQVED | 2,679 | 2,454 | B |
| PAAG_06380 | pyruvate kinase | 307 | QKMMIAKCNIKGKPV | 23,204 | 20,743 | C |
| PAAG_06380 | pyruvate kinase | 316 | IKGKPVICATQMLES | 3,804 | 2,454 | B |
| PAAG_06380 | pyruvate kinase | 348 | AVLDGADCVMLSGET | 20,818 | 20,743 | C |
| PAAG_06380 | pyruvate kinase | 372 | VMMMHETCLIAEVAI | 22,38 | 20,743 | C |
| PAAG_02050 | pyruvate decarboxylase | 143 | NMSASISCYVARLNP | 24,095 | 20,743 | C |
| PAAG_04559 | 2-methylcitrate dehydratase | 398 | TIRTHEACVRIIDKQ | 2,565 | 2,454 | B |
| PAAG_01725 | succinate dehydrogenase flavoprotein subunit | 35 | AVASELTCSIFQFTK | 24,175 | 20,743 | C |
| PAAG_01725 | succinate dehydrogenase flavoprotein subunit | 113 | EAGFNTACISKLFPT | 2,598 | 2,454 | B |
| PAAG_01725 | succinate dehydrogenase flavoprotein subunit | 329 | TGIYGAGCLITEGSR | 20,745 | 20,743 | C |
| PAAG_01725 | succinate dehydrogenase flavoprotein subunit | 599 | ELRNLLTCATQTAIA | 3,38 | 2,454 | B |
| PAAG_00856 | isocitrate dehydrogenase | 15 | QSAQSLFCTIAKNSS | 23,81 | 20,743 | C |
| PAAG_01534 | pyruvate dehydrogenase E1 component subunit β | 32 | LAFRPAVCAPSALQR | 3,663 | 2,454 | B |
| PAAG_01534 | pyruvate dehydrogenase E1 component subunit β | 111 | PITEAGFCGLAVGAA | 20,818 | 20,743 | C |
| PAAG_01534 | pyruvate dehydrogenase E1 component subunit β | 263 | TIVSLSRCVGQAIAA | 3,478 | 2,454 | B |
| PAAG_03330 | dihydrolipoyl dehydrogenase | 24 | QMRQSQICSASAVLS | 3,027 | 2,454 | B |
| PAAG_05518 | cell division cycle protein | 225 | GYDDIGGCRKQMAQI | 3,13 | 2,454 | B |
| PAAG_03532 | actin | 2 | MCKAGFAGD | 4,23 | 1,67 | A |
| PAAG_03532 | actin | 202 | RDIKEKLCYVALDFQ | 22,416 | 20,743 | C |
| PAAG_03532 | actin | 270 | TYNAIMKCDVDVRKD | 3,31 | 2,454 | B |
| PAAG_03816 | 40S ribosomal protein S4 | 41 | GPHKLRDCLPLVVFI | 2,533 | 2,454 | B |
| PAAG_03816 | 40S ribosomal protein S4 | 124 | EEAEYKLCKVKRVQL | 22,978 | 20,743 | C |
| PAAG_08634 | 40S ribosomal protein S12 | 132 | RKVVNCSCVVLRDWG | 21,62 | 20,743 | C |
| PAAG_05704 | 40S ribosomal protein S13 | 38 | EQVVDQICKLAKKGA | 22,774 | 20,743 | C |
| PAAG_06487 | 60S ribosomal protein L7 | 195 | LGKFGIVCMEDLIHE | 3,462 | 2,454 | B |
| PAAG_01052 | 60S ribosomal protein L10 | 59 | ALEAARICANKYLVK | 2,739 | 2,454 | B |
| PAAG_01052 | 60S ribosomal protein L10 | 93 | RINKMLSCAGADRLQ | 2,625 | 2,454 | B |
| PAAG_00689 | ATP-dependent RNA helicase eIF4A | 398 | PMNVAGMC | 2,011 | 1,67 | A |
| PAAG_01727 | T-complex protein 1 subunit delta | 289 | KKIAKTKCNVLLIQK | 22,504 | 20,743 | C |
| PAAG_01727 | T-complex protein 1 subunit delta | 405 | RSLHDALCVIRCLVK | 21,358 | 20,743 | C |
| PAAG_07444 | hsp70 | 16 | SSESGGRCAIGLSFG | 20,752 | 20,743 | C |
| PAAG_12063 | protein transporter SEC23 | 355 | IVDIFAGCLDQVGLL | 2,707 | 2,454 | B |
| PAAG_12063 | protein transporter SEC23 | 442 | NSVGETECGIGNTCS | 23,007 | 20,743 | C |
| PAAG_05019 | hypothetical protein | 8 | MAAAVAACIFCKIVK | 3,552 | 1,67 | A |
| PAAG_05019 | hypothetical protein | 11 | AVAACIFCKIVKGDI | 20,774 | 20,743 | C |
| PAAG_04609 | mis6 domain-containing protein | 101 | TVSKVISCFGPGKSK | 2,538 | 2,454 | B |
| PAAG_04609 | mis6 domain-containing protein | 670 | LCRASSACFGRIEEE | 2,87 | 2,454 | B |
| PAAG_01842 | β-lactamase family protein | 9 | GKDISPECVSLIRQA | 4,321 | 2,454 | B |
| PAAG_01842 | β-lactamase family protein | 21 | RQALEDACADQERGI | 1,842 | 1,67 | A |
| PAAG_11068 | hypothetical protein | 71 | QLNMQGACEISRPEL | 22,321 | 20,743 | C |
| PAAG_11068 | hypothetical protein | 83 | PELKQLRC | 3,418 | 2,454 | B |

1 A physicochemical and statistical value for each position surrounding nitrosylation sites.

^2^ A value where strong dependence is detected, with p = 0.005 and 16 degrees of freedom between two positions.

^3^ Classification of the positive sets into tree-like subgroups.
